# Computer-Aided Rational Design of Efficient NADPH Production System by *Escherichia coli pgi* Mutant Using a Mixture of Glucose and Xylose

**DOI:** 10.3389/fbioe.2020.00277

**Published:** 2020-04-07

**Authors:** Yu Matsuoka, Hiroyuki Kurata

**Affiliations:** ^1^Department of Bioscience and Bioinformatics, Kyushu Institute of Technology, Iizuka, Japan; ^2^Biomedical Informatics R&D Center, Kyushu Institute of Technology, Iizuka, Japan

**Keywords:** kinetic modeling, co-consumption of glucose and xylose, carbon catabolite regulation, multiple carbon source, Pgi mutant, NADPH over-production, *Escherichia coli*

## Abstract

Lignocellulosic biomass can be hydrolyzed into two major sugars of glucose and xylose, and thus the strategy for the efficient consumption of both sugars is highly desirable. NADPH is the essential molecule for the production of industrially important value-added chemicals, and thus its availability is quite important. *Escherichia coli* mutant lacking the *pgi* gene encoding phosphoglucose isomerase (Pgi) has been preferentially used to overproduce the NADPH. However, there exists a disadvantage that the cell growth rate becomes low for the mutant grown on glucose. This limits the efficient NADPH production, and therefore, it is quite important to investigate how addition of different carbon source such as xylose (other than glucose) effectively improves the NADPH production. In this study, we have developed a kinetic model to propose an efficient NADPH production system using *E. coli pgi*-knockout mutant with a mixture of glucose and xylose. The proposed system adds xylose to glucose medium to recover the suppressed growth of the *pgi* mutant, and determines the xylose content to maximize the NADPH productivity. Finally, we have designed a mevalonate (MVA) production system by implementing ArcA overexpression into the *pgi*-knockout mutant using a mixture of glucose and xylose. In addition to NADPH overproduction, the accumulation of acetyl-CoA (AcCoA) is necessary for the efficient MVA production. In the present study, therefore, we considered to overexpress ArcA, where ArcA overexpression suppresses the TCA cycle, causing the overflow of AcCoA, a precursor of MVA. We predicted the xylose content that maximizes the MVA production. This approach demonstrates the possibility of a great progress in the computer-aided rational design of the microbial cell factories for useful metabolite production.

## Introduction

Microbial production of biofuels and biochemicals from renewable feedstocks has received considerable attention from environmental protection and energy production perspectives. Lignocellulosic biomass is regarded as the most promising feedstock, because it is abundant and lower carbon footprint (Valdehuesa et al., 2018). Since the lignocellulosic biomass can be hydrolyzed into two major sugars of glucose and xylose (Gawand et al., 2013), the strategy for the efficient consumption of both sugars is highly desirable for the production of useful chemicals with low cost. In the production of industrially important value-added products such as 3-hydroxybutyrate (3HB), methyl 3-hydoxybutyrate (MHB), amino acids, fatty acids, and isoprenoids (Siedler et al., 2011; Perez-Zabaleta et al., 2016; Yanase et al., 2016; Niu et al., 2017; Li et al., 2018), NADPH is the essential molecule, and thus its availability remains a major hurdle for the efficient production of useful chemicals and fuels (Spaans et al., 2015). Several metabolic engineering strategies have, therefore, been considered, and tested in practice.

Although NADPH can be produced at several metabolic sites such as the oxidative pentose phosphate (PP) pathway via glucose 6-phosphate dehydrogenase (G6PDH) and 6-phosphogluconate dehydrogenase (6PGDH), as well as isocitrate dehydrogenase (ICDH) in the TCA cycle, and malic enzyme (Mez), the oxidative PP (OPP) pathway is the dominant site in many organisms including *Escherichia coli* (Gvozdev et al., 1976; Jan et al., 2013). The typical approach for the overproduction of NADPH is, therefore, to increase the flux through the OPP pathway by the disruption of *pgi* gene encoding phosphoglucose isomerase (Pgi) (and also *pfkA* encoding phosphofructokinase (Pfk) but to a lesser extent), which forces the flux of the imported glucose toward the OPP pathway at the glucose 6-posphate (G6P) node, which resulted in increased NADPH. In fact, this strategy has been employed to produce several chemicals in practice (Kabir and Shimizu, 2003; Marx et al., 2003; Blombach et al., 2008; Wang et al., 2013; Park et al., 2014; Seol et al., 2014; Kim et al., 2015; Ng et al., 2015; Aslan et al., 2017; Sundara Sekar et al., 2017). Since excess NADPH allosterically inhibits the activity of G6PDH, G6P accumulates and the glucose uptake rate (GUR) reduces through the post-transcriptional regulation, causing instability of mRNA of *ptsG* encoding EIIBC of the phosphotransferase system (PTS) (Morita et al., 2003). Therefore, the cell growth rate of the *pgi* mutant becomes significantly lower (by about 80 %) than that of the wild-type strain when glucose was used as a carbon source (Charusanti et al., 2010). In order to avoid the lower cell growth rate inherent in the *pgi*-knockout mutant, several attempts have been made by partially increasing the Pgi activity (Usui et al., 2012), by reducing the expression level of its gene via replacement of its start codon ATG with GTG without completely removing Pgi (Park et al., 2014; Kim et al., 2015), and by over-expressing the genes of the OPP pathway (Lim et al., 2002; Perez-Zabaleta et al., 2016).

It has been shown that the deletion of the *pgi* gene affects the physiological and metabolic changes depending on the carbon sources (Fraenkel and Levisohn, 1967). In the case of using fructose as a carbon source, the cell growth rate of the *pgi* mutant is not much affected (unlike the case of using glucose), but the NADPH production is totally shut down at the OPP pathway and is limited only at ICDH and Mez reactions (Ahn et al., 2011). With this in mind, the effect of co-consumption of glucose and another carbon source such as fructose or xylose on the production of useful chemicals by the *E. coli pgi* mutant has been investigated (Ahn et al., 2011; Wang et al., 2018). It has been shown that the co-consumption of glucose and xylose can be attained in the *pgi*-deficient mutant, resulting in the higher cell growth rate and methyl-ketone production as compared to the case of using glucose as a carbon source (Wang et al., 2018). Although an attempt has been made to characterize the experimentally observed metabolic states, it is difficult for *in silico* constraints-based flux analysis to predict the intracellular metabolic behavior under various experimental conditions without incorporating the metabolic regulation systems (Ahn et al., 2011). The carbon catabolite repression (CCR) plays an essential role in the co-consumption of multiple carbon sources (Kim et al., 2010), and the metabolic regulation analysis is necessary for the efficient microbial cell factories and the optimization of the culture condition. Namely, it is necessary to analyze the intracellular metabolism based on the metabolic regulation mechanisms by the kinetic modeling approach (Matsuoka and Shimizu, 2013; Jahan et al., 2016; Kurata and Sugimoto, 2017; Matsuoka and Kurata, 2017; Millard et al., 2017). Of the various modeling approaches currently available, the kinetic modeling approach is promising for realizing the essential feature of the metabolic regulation because it can describe the complex reactions such as allosteric modulation (Link et al., 2013), enzyme modification (Kremling et al., 2001), and gene expression by transcription factors (TFs) (Hardiman et al., 2007; Henkel et al., 2014).

In this study, we have developed a kinetic model to design the efficient NADPH production system by an *E. coli pgi*-knockout mutant using a mixture of glucose and xylose, since NADPH is essential for producing industrially important chemicals. We used the proposed efficient NADPH production system to rationally design an ArcA-overexpressing, *pgi*-knockout mutant using glucose and xylose as carbon sources for enhanced mevalonate (MVA) production.

## Materials and Methods

### Modeling the Central Carbon Metabolism

**FIGURE 1:**
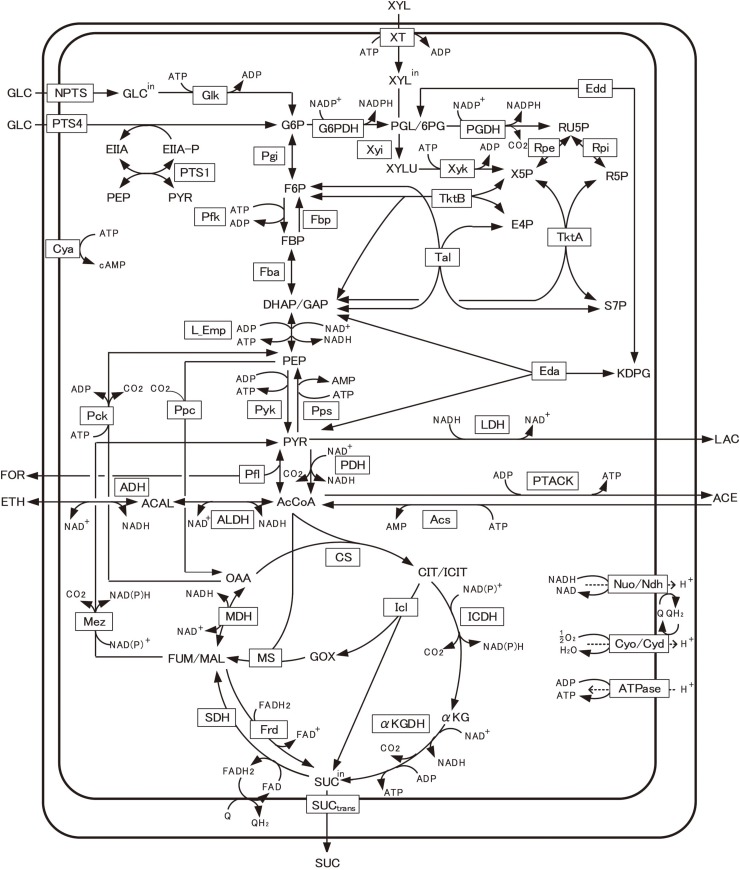
Main metabolic pathways in *Escherichia coli*. The present model includes glycolysis, TCA cycle, respiratory chain, pentose phosphate (PP), gluconeogenic, glyoxylate, anaplerotic, fermentative, and xylose assimilating pathways, as well as the glucose transport system of phosphotransferase system (PTS) and xylose transport (XT). The full name of the metabolite and enzyme is given in Nomenclature. Transcriptional regulations are given in Supplementary Table S1.

Figure 1 shows the primary metabolic pathways of *E. coli*, including glycolysis, TCA cycle, respiratory chain, pentose phosphate (PP), gluconeogenic, glyoxylate, anaplerotic, fermentative, and xylose assimilating pathways, as well as the substrate transport systems such as PTS and xylose transport (XT). The detailed mass balance equations and the kinetic model equations are given in Supplementary Material.

Once the overall metabolic fluxes of the primary metabolism are calculated, the specific ATP production rate can be estimated. Referring to Figure 1, the specific ATP production rate can be expressed as follows:

(1) $$v_{A\,T\,P}=O\,P+v_{L\,\_\,E\,m\,p}+v_{P\,y\,k}+v_{P\,T\,A\,C\,K}+v_{\alpha \,K\,G\,D\,H}-v_{G\,l\,k}-v_{P\,f\,k}-v_{P\,p\,s}-v_{P\,c\,k}-v_{A\,c\,s}-v_{X\,T}-v_{X\,y\,k}$$

ATP is produced via either substrate-level phosphorylation or oxidative phosphorylation. Note that L_Emp is the lumped pathway from glyceraldehyde-3-phosphate/dihydroxy acetone phosphate (GAP/DHAP) to PEP, and PTACK is the combined pathway for Pta and Ack (Figure 1). In Eq. (1), OP represents the specific ATP production rate via oxidative phosphorylation (Matsuoka and Kurata, 2017), as described in Supplementary Material.

The specific growth rate (μ) was estimated based on the experimental observation that the cell growth and the specific ATP production rates are linearly correlated (Nanchen et al., 2006; Kadir et al., 2010; Yao et al., 2011) such that:

(2) $$\mu =k_{A\,T\,P}\cdot v_{A\,T\,P}$$

where *k*_*ATP*_ represents the constant parameter, and *v*_*ATP*_ represents the specific ATP production rate computed by Eq. (1).

The hallmark of the present model is that the metabolic regulation mechanisms are incorporated. Enzyme level regulation can be represented by incorporating the effectors (metabolites) into the corresponding kinetic models. Transcriptional regulation is also important and can be represented by the TFs (Matsuoka and Kurata, 2017), such that:

(3) $$v_{•}^{\max}=v_{•}^{\max,\prime }\cdot f\,\left(T\,F_{i}\right)$$

where *TF*_*i*_ represents the activity of the *i*th transcription factor, and $v_{•}^{\max,\prime }$ represents the original maximum reaction rate for the corresponding pathway reaction. The detailed equations are given in Supplementary Material. Supplementary Table S1 shows the effects of TFs on the primary metabolic pathways included in the present model. A “+” sign represents the case in which the TF activates gene expression, whereas a “–” sign represents the case in which the TF represses gene expression. The gene name is written in brackets, where *npts* denotes the gene that codes for glucose transporters other than glucose-PTS, and *L_emp* denotes a hypothetical gene that codes for the lumped reactions through glyceraldehyde-3-phosphate dehydrogenase (GAPDH), phosphoglucokinase (Pgk), phosphoglucomutase (Pgm), and enolase (Eno).

We have extended our previous model by incorporating the xylose metabolism and its regulations as follows:

(4a) $$\frac{\text{d}\,\left[X\,Y\,L^{i\,n}\right]}{\text{d}\,t}=v_{X\,T}-v_{X\,y\,i}-\mu \,\left[X\,Y\,L^{i\,n}\right]$$

(4b) $$\frac{\text{d}\,\left[X\,Y\,L\,U\right]}{\text{d}\,t}=v_{X\,y\,i}-v_{X\,y\,k}-\mu \,\left[X\,Y\,L\,U\right]$$

where *v*_*XT*_ represents the XT for xylose consumption. Since the unphosphorylated EIIA prevents the uptake of carbon sources excluding glucose (inducer exclusion), we expressed its equation as the expression of *v*_*NPTS*_ (Bettenbrock et al., 2006) as follows:

(5a) $$v_{X\,T}=\frac{v_{X\,T}^{\max,\prime }\cdot f\,\left(T\,F_{c\,A\,M\,P-C\,r\,p},T\,F_{X\,y\,l\,R}\right)\cdot \left[X\,Y\,L\right]}{K_{X\,Y\,L}+\left(1+\frac{\left[E\,I\,I\,A\right]}{K_{I}}\right)\,\left[X\,Y\,L\right]}$$

where *f*(*TF*_*cAMP*−*Crp*_,*TF*_*XylR*_) represents the transcriptional regulation for the XT of which gene expression is regulated by cAMP-Crp and XylR. The equations for *v*_*Xyi*_ and *v*_*Xyk*_ were expressed as follows (Altintas et al., 2006):

(5b) $$v_{X\,y\,i}=\frac{v_{X\,y\,i}^{\max,\prime }\cdot f\,\left(T\,F_{c\,A\,M\,P-C\,r\,p},T\,F_{X\,y\,l\,R}\right)\cdot \left[X\,Y\,L^{i\,n}\right]}{K_{X\,Y\,L^{i\,n}}+\left[X\,Y\,L^{i\,n}\right]}$$

(5c) $$v_{X\,y\,k}=\frac{v_{X\,y\,k}^{\max,\prime }\cdot f\,\left(T\,F_{c\,A\,M\,P-C\,r\,p},T\,F_{X\,y\,l\,R}\right)\cdot \left[X\,Y\,L\,U\right]\,\left[A\,T\,P\right]}{K_{X\,Y\,L\,U}\,K_{A\,T\,P}+K_{A\,T\,P}\,\left[X\,Y\,L\,U\right]+K_{X\,Y\,L\,U}\,\left[A\,T\,P\right]+\left[X\,Y\,L\,U\right]\,\left[A\,T\,P\right]}$$

where xylose assimilating pathway genes are also regulated by cAMP-Crp and XylR.

### Simulation Condition for Model Construction

For the model construction, the wild-type (WT) strain, *pfl*-, and *ack*- knockout mutants of *E. coli* were simulated in batch cultures under both anaerobic and aerobic conditions using glucose and xylose as a carbon source. To compare the simulated fluxes with the experimental fluxes, 40 mM glucose and 40 mM xylose were used in the WT strain, as well as experimental condition (Gonzalez et al., 2017). To compare the simulated growth rates with the experimental ones, 3 g/l glucose and 3 g/l xylose were used in the WT strain, *pfl*-, and *ack*- knockout mutants under aerobic condition, while 10 g/l glucose and 10 g/l xylose with 1 g/l acetate were used under anaerobic condition, as well as experimental condition (Hasona et al., 2004). Moreover, the simulated metabolic fluxes of a *ptsG*-knockout mutant were compared with the experimental data obtained in the aerobic batch culture using a mixture of 10 mM glucose and 10 mM xylose (Long et al., 2016). In addition, the WT strain and single-gene mutants (for the glycolytic and PP pathway genes) were simulated to validate the intracellular metabolic fluxes in the continuous cultures, where the dilution rates were 0.1, 0.2, 0.4, and 0.5 h^–1^ for the WT strain and 0.2 h^–1^ for the single-gene mutants under aerobic condition, where the concentration of the feeding glucose was 4 g/l, in accordance with the experimental condition (Ishii et al., 2007).

### Simulation Condition for Prediction

For investigating the metabolic regulation mechanisms of the multi-carbon metabolism, WT strain, *pgi*-, and *ptsG*- knockout mutants of *E. coli* were simulated in the batch cultures under aerobic condition using 4 g/l glucose or a mixture of 5 g/l glucose and 5 g/l xylose as a carbon source, in accordance with the experimental conditions (Toya et al., 2010; Matsuo, 2011). To analyze the efficient production of NADPH, such strains were further simulated by varying the xylose content, where the total substrate concentration of glucose and xylose was set to 10 g/l.

The MVA-producing strain of *E. coli* was simulated in the batch culture under aerobic condition for the case of using 4 g/l glucose as a carbon source, in accordance with the experimental condition (Wada et al., 2017). The *pgi* mutant with the overexpression of ArcA was simulated to enhance the MVA production, where the total concentration of glucose and xylose was set to 4 g/l.

### Numerical Simulation

All the ordinary differential equations were numerically solved by ode15s (Matlab).

### Evaluating the NADPH Produced in Batch Cultivation

Since the cell concentration changes with respect to time, the total NADPH produced during the batch culture can be expressed as,

(6) $$N\,A\,D\,P\,H\,p\,r\,o\,d\,u\,c\,t\,i\,o\,n\equiv \int_{0}^{T}v_{N\,A\,D\,P\,H}\,\left(t\right)\cdot X\,\left(t\right)\,dt$$

where *v*_*NADPH*_(*t*) and *X*(*t*) are the specific NADPH production rate and the cell concentration at time *t*, respectively, and *T* represents the cultivation time. The specific NADPH production rate is expressed as follows:

(7) $$v_{N\,A\,D\,P\,H}\,\left(t\right)=v_{G\,A\,P\,D\,H}\,\left(t\right)+v_{P\,G\,D\,H}\,\left(t\right)+v_{I\,C\,D\,H}\,\left(t\right)+v_{M\,e\,z}\,\left(t\right)$$

To achieve the efficient NADPH production with low cost, the productivity of NADPH is quite important, where it is calculated by dividing the available NADPH by *T* as follows:

(8) $$N\,A\,D\,P\,H\,p\,r\,o\,d\,u\,c\,t\,i\,v\,i\,t\,y\,\equiv \frac{1}{T}\,\left\{\int_{0}^{T}v_{N\,A\,D\,P\,H}\,\left(t\right)\cdot X\,\left(t\right)\,dt-C_{A\,n\,a\,b\,o\,l\,i\,s\,m}\right\}$$

where *C*_*Anabolism*_ is the total amount of NADPH to be consumed for the biomass formation during the batch culture, and it is expressed as follows:

(9) $$C_{A\,n\,a\,b\,o\,l\,i\,s\,m}\equiv k_{A\,n\,a\,b\,o\,l\,i\,s\,m}\cdot X\,\left(T\right)$$

where *k*_*Anabolism*_ is the amount of NADPH required for the cell synthesis (per cell), which has been identified to be 14.849 mmol/gDCW (Marx et al., 1996) and *X*(*T*) is the cell concentration at time *T*, namely, the final cell concentration.

## Results and Discussion

### Model Construction of Multi-Carbon Metabolism

We have developed a kinetic model of multi-carbon metabolism for the *E. coli* central metabolic pathway for the case of using a mixture of glucose and xylose as a carbon source (Figure 1). Model parameters of the kinetic rate equations were identified by using the experimental (literature) data as the training data. All parameter values are listed in Supplementary Tables S2–S4. The training data used are four independent experimental data of the intracellular metabolic fluxes of WT strain in the batch cultures under both anaerobic and aerobic conditions using glucose and xylose as a carbon source (Gonzalez et al., 2017). Model parameters were adjusted so that the model can reproduce the experimental data. The experimental data (Gonzalez et al., 2017) were compared with the simulation results as shown in Supplementary Figure S1. The correlation coefficients between the measured and simulated metabolic fluxes were 0.993 and 0.940 under aerobic batch cultivations using glucose and xylose, respectively. The correlation coefficients between both the fluxes were 0.992 and 0.970 under anaerobic batch cultivations using glucose and xylose, respectively. All *p*-values were less than 0.01. Thus, the simulated metabolic fluxes represent well the experimental data.

The present model was validated by the three test datasets. The first one is the dataset of the experimental cell growth rates of WT, *pfl*-, and *ack*- knockout mutants grown on glucose and xylose for the batch cultures under both anaerobic and aerobic conditions (Hasona et al., 2004). The correlation coefficients between the measured and simulated growth rates were 0.962 (*p* < 0.01), as shown in Supplementary Figure S2. The present model reproduced the experimental data of the intracellular fluxes and cell growth in the WT strain under anaerobic condition for the case of using glucose as a carbon source (Supplementary Figures S1C, S2). While the kinetic model reproduced the intracellular fluxes (Supplementary Figure S1D), the simulated growth rate was underestimated in the case of using xylose as a carbon source under anaerobic condition (Supplementary Figure S2). It is because the kinetic model does not incorporate complex ATP consumption mechanisms responsible for the xylose uptake (via multiple transporters) due to its uncertainty.

The second is the dataset of the intracellular metabolic fluxes of the *ptsG*-knockout mutant grown on glucose and xylose for the batch culture under aerobic condition (Long et al., 2016). The correlation coefficient between the measured and simulated fluxes was 0.956 (*p* < 0.01), as shown in Supplementary Figure S3. The third is the datasets of the intracellular metabolic fluxes in the continuous cultures. The present model reproduced the intracellular metabolic fluxes of the WT and single-gene knockout mutants grown on glucose in continuous cultures (Ishii et al., 2007), as shown in Supplementary Table S5.

### Dynamics on Glucose in Aerobic Batch Culture

Supplementary Figure S4 shows the time-profiles of aerobic batch cultures of the WT strain (Supplementary Figure S4A), *pgi*-knockout mutant (Supplementary Figure S4B), and *ptsG*-knockout mutant (Supplementary Figure S4C) grown on glucose, where the lines and symbols represent the simulation results and the experimental data (Toya et al., 2010), respectively. It has been reported that the deletion of the *ptsG* gene relaxes the CCR (Luo et al., 2014), which enables the co-consumption of multiple carbon sources, as well as the *pgi*-knockout mutant (Ahn et al., 2011). The simulated time-course profile for the *ptsG*-knockout mutant also represents the experimental data (Matsuo, 2011), as shown in Supplementary Figure S5.

Supplementary Figures S4B,C indicate that the GURs (as well as the cell growth rates) of *pgi*- and *ptsG*- knockout mutants are significantly lower than the GUR of the WT strain (Supplementary Material). As implied from Supplementary Figure S4D, the main reason for the lower GUR of the *pgi* mutant is the accumulation of glucose 6-phosphate (G6P), as experimentally observed (Toya et al., 2010). The increased G6P pool size causes destabilization of the mRNA of *ptsG* gene, decreasing the GUR (Negrete and Shiloach, 2017), while a large portion of glucose is consumed by the alternative (less-efficient) glucose transporters such as GalP, ManII, and MglCAB (termed as NPTS) (Supplementary Figure S4D). Due to the lower PTS flux, the PEP/PYR ratio in the *pgi* and *ptsG* mutants are higher than that in the WT strain, which causes higher activities of cAMP-Crp than that of the WT strain (Supplementary Figure S4D). This results in the activation of the non-PTS fluxes (v_NPTS_) in the *pgi* and *ptsG* mutants (Supplementary Figure S4D). In the *pgi* (and *ptsG*) mutant, the pool size of FBP decreases (Supplementary Figure S4D), which allosterically deactivates the Pyk activity. The decrease in FBP also causes Cra activity to increase (Supplementary Figure S4D), which transcriptionally represses the expression of *pykF* gene encoding Pyk. Thus, the Pyk flux of the *pgi* mutant was lower than that of the WT strain (Supplementary Figure S4D). As seen above, the metabolic regulation mechanisms are transparent based on the kinetic modeling to explain the fermentation data.

### Dynamics on a Mixture of Glucose and Xylose in Aerobic Batch Culture

**FIGURE 2:**
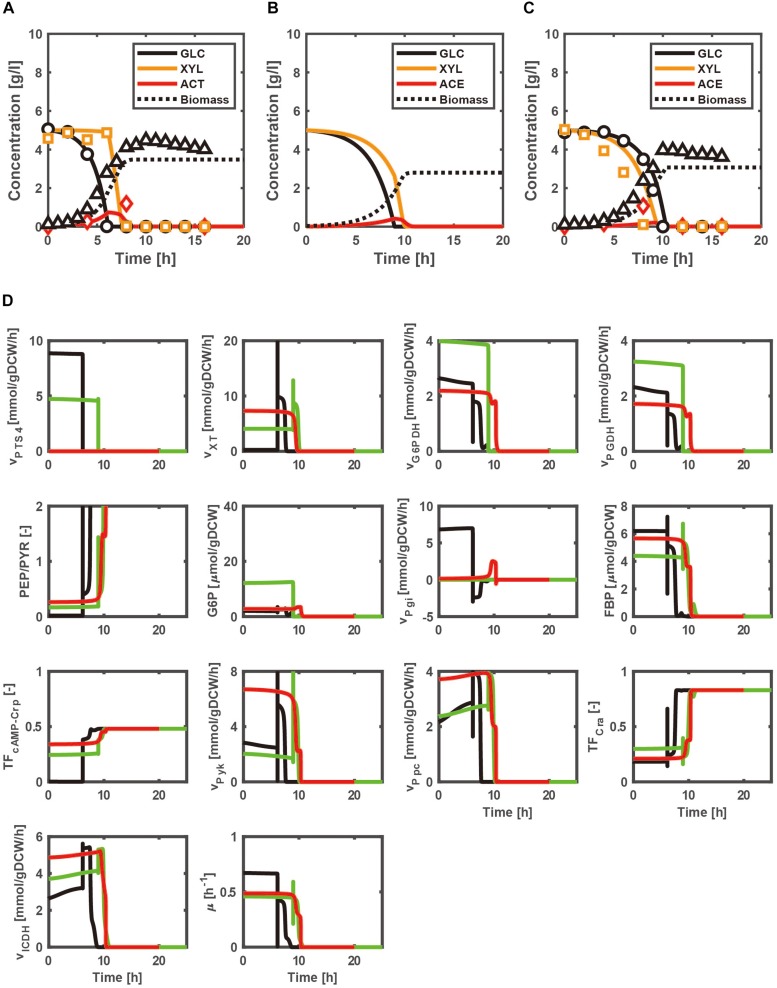
Simulation results of the batch cultivations of the wild type, and *pgi*- and *ptsG*- knockout mutants grown on a mixture of glucose and xylose under aerobic condition. The lines and symbols represent the simulated and experimental (Matsuo, 2011) data for the time courses of extracellular metabolites and biomass concentrations of wild type **(A)**, and *pgi*- **(B)** and *ptsG*- **(C)** knockout mutants, respectively. The black, green, and red lines represent the simulated time courses of the intracellular metabolic changes for the cases of the wild type, *pgi* mutant, and *ptsG* mutant, respectively **(D)**.

Figures 2A–C show the time courses of the extracellular metabolites and biomass concentrations for the batch cultures of the WT strain, and *pgi*- and *ptsG*- knockout mutants grown on a mixture of glucose and xylose as a carbon source under aerobic condition. Figure 2A indicates that glucose is first consumed in the WT strain, while xylose is consumed after glucose is depleted due to CCR. On the other hand, co-consumption of multiple carbon sources is attained in the *pgi* and *ptsG* mutants (Figures 2B,C), due to the relaxed CCR caused by the increased cAMP-Crp activities (Figure 2D). Comparison of Figures 2A–C with the respective Supplementary Figures S4A–C indicates that the carbon source uptake rates as well as the cell growth rates of *pgi* and *ptsG* mutants increase for the case of using a mixture of glucose and xylose as compared to the case of using only glucose.

As compared with Supplementary Figure S4D, the FBP concentration increases with xylose addition in the *pgi* mutant, which decreases Cra activity to increase the OPP pathway fluxes (G6PDH and PGDH fluxes) (Figure 2D). This causes the decrease in the G6P accumulation, improving the GUR (Figure 2B). The xylose uptake rate is enhanced in the *pgi* and *ptsG* mutants due to cAMP-Crp activity being higher than that of the WT strain during the batch culture (Figure 2D). As shown in Figures 2B,C, the carbon source uptake becomes faster than that for the case of using only glucose (Supplementary Figures S4B,C), partly due to higher cell growth rate. Namely, the specific growth rates in the *pgi* and *ptsG* mutants using multiple carbon sources increase as compared to the case of using glucose alone, because the glycolytic and TCA cycle fluxes increase in the *pgi* and *ptsG* mutants using multiple carbon sources (Figure 2D and Supplementary Figure S4D).

**FIGURE 3:**
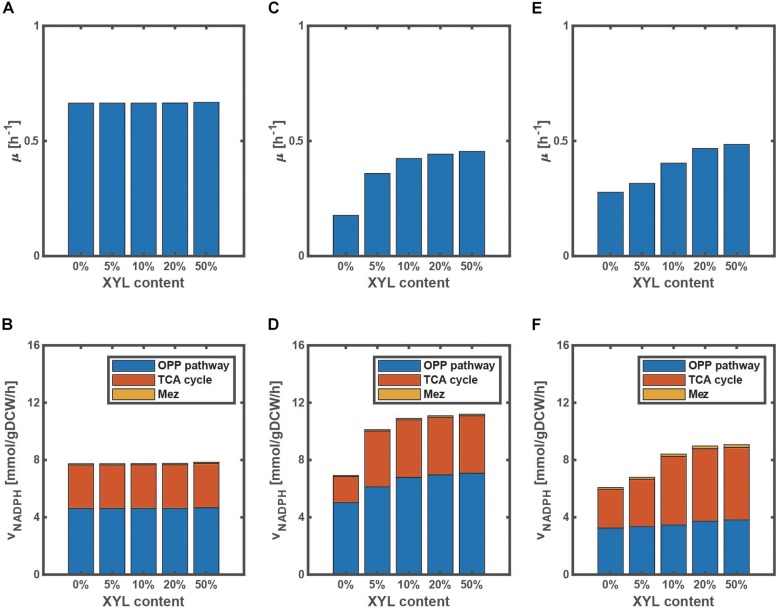
Effect of the change in xylose content on the specific growth rate and the specific NADPH production rate during the exponential phase in wild type **(A,B)**, *pgi* mutant **(C,D)**, and *ptsG* mutant **(E,F)** grown on a mixture of glucose and xylose. The xylose content [%] is defined as the ratio of xylose contained in a mixture. The total substrate concentration is 10 g/l.

In fact, the extent of the improvement depends on the ratio of the mixture as illustrated in Figure 3, where the specific cell growth rate and the specific NADPH production rate [as expressed by Eq. (7) during the exponential phase] are shown for the WT strain (Figures 3A,B), *pgi* mutant (Figures 3C,D), and *ptsG* mutant (Figures 3E,F). Since glucose is preferentially consumed due to CCR in the WT strain, the cell growth rate does not change even if the xylose content increases (Figure 3A), where the xylose content [%] is defined as the ratio of xylose contained in a mixture of glucose and xylose. On the other hand, the cell growth rates of the *pgi* and *ptsG* mutants increase as the xylose content increases (Figures 3C,E). The specific NADPH production rate depends on the xylose content in the *pgi* and *ptsG* mutants (Figures 3D,F). The specific NADPH production rate by OPP pathway (v_G6PDH_+v_PGDH_) is dominant in the *pgi* mutant, while the production rate by TCA cycle (v_ICDH_) is rather dominant in the *ptsG* mutant, as also seen in Figure 2D.

It has been reported that the evolved *pgi* mutant recovered the cell growth rate, due mainly to mutation of transhydrogenase such as UdhA (as well as Pnt) (Long et al., 2018), thus relaxing the inhibition of G6PDH by NADPH. Note that this evolutional experiment was conducted using glucose as a carbon source. If xylose is available in addition to glucose, the cell growth rate can be also improved as shown by Figure 3C.

### Effect of Xylose Content on NADPH Availability and Productivity

**FIGURE 4:**
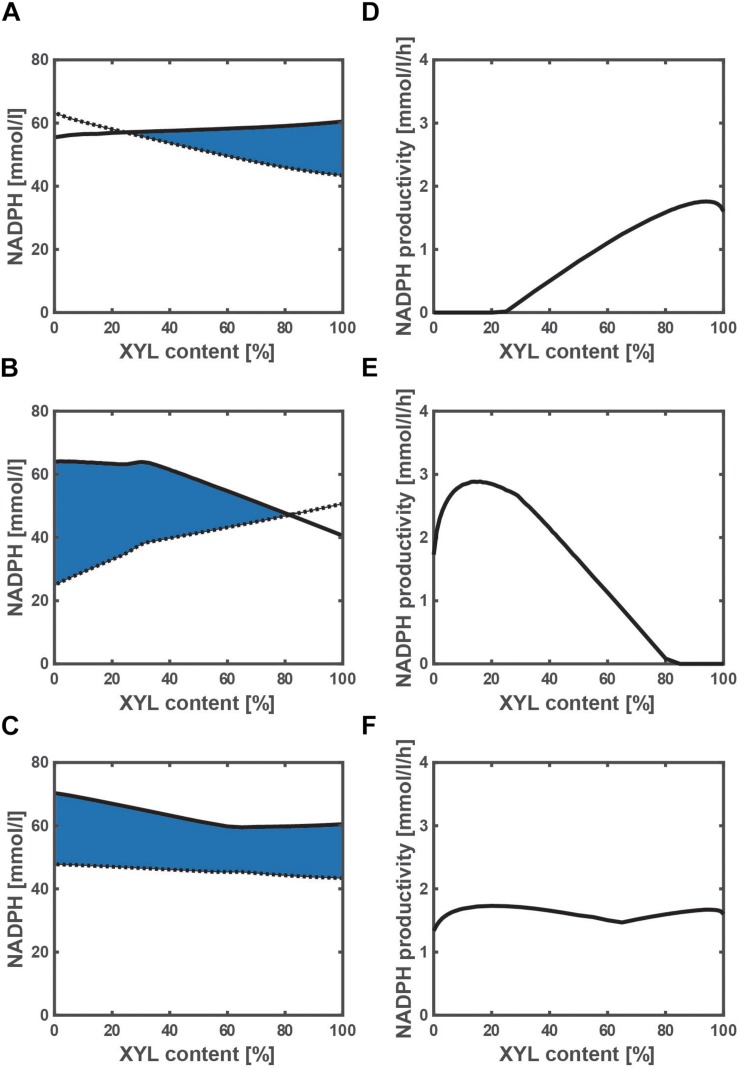
Effect of the change in xylose content on the NADPH availability and NADPH productivity in wild type **(A,D)**, *pgi* mutant **(B,E)**, and *ptsG* mutant **(C,F)** grown on a mixture of glucose and xylose. **(A–C)** The solid and dotted lines represent the total NADPH production as expressed by Eq. (6) and the NADPH required for the biosynthesis as expressed by Eq. (9), respectively. The area surrounded by the solid and dotted lines as colored in blue is the extra NADPH that can be used for the useful metabolite production (i.e., NADPH availability). The xylose content [%] is defined as the ratio of xylose contained in a mixture of glucose and xylose. The total substrate concentration is 10 g/l.

To investigate the effect of a change in xylose content on the NADPH availability and productivity, we simulated the WT strain and the two mutants grown on a mixture of glucose and xylose. NADPH is essential for the cell synthesis (anabolism), and the required NADPH for the cell synthesis as expressed by Eq. (9) is shown by the dotted line in Figures 4A–C. The final cell concentration varies with the xylose content (Supplementary Figure S6), because the cell growth rate depends on a carbon source of either glucose or xylose. Since glucose is the preferred carbon source as compared to xylose in the WT strain, the final cell concentration decreases as the xylose content increases (Supplementary Figure S6A), and thus the dotted line decreases accordingly (Figure 4A). On the other hand, the final cell concentration increases as the xylose content increases in the *pgi* mutant (Supplementary Figure S6B), and thus the dotted line increases accordingly (Figure 4B). This opposing trend is caused by the fact that the inhibition of the glucose uptake by the accumulated G6P is relaxed as the xylose content increases. Unlike the WT strain and *pgi* mutant, the final cell concentration hardly changes with the xylose content in the *ptsG* mutant (Supplementary Figure S6C), and the trend is the same as the dotted line in Figure 4C.

The total NADPH produced during the batch cultivation [as expressed by Eq. (6)] is shown by the solid line in Figures 4A–C with respect to the xylose content. As shown in Figure 4A, the NADPH produced is less than that required for the cell growth at higher glucose content (or lower xylose content), and thus NADPH may need to be backed up by transhydrogenase PntAB from NADH in the WT strain. In fact, the fraction of NADPH produced via Pnt increases as the cell growth rate increases (Sauer et al., 2004). On the other hand, the NADPH produced is less than that required for the cell growth at higher xylose content (Figure 4B). In the case of the *pgi* mutant, the flux from fructose 6-phosphate (F6P) to G6P is blocked, and thus any carbon source such as xylose that is introduced to F6P cannot produce NADPH at the OPP pathway. Therefore, the shortage of NADPH for the cell growth needs to be backed up by Pnt, as well as the case of using fructose as a carbon source (Ahn et al., 2011).

The extra NADPH that can be used for the useful metabolite production (i.e., NADPH availability) can be computed by subtracting the NADPH necessary for the cell growth from the total NADPH produced, which is the area surrounded by the solid and dotted lines as colored in blue (Figures 4A–C). The NADPH availability of the *pgi* mutant grown on only glucose is the highest among three strains. However, the cultivation time is prolonged in the *pgi* mutant using glucose as a carbon source, since the cell growth rate is low as shown in Supplementary Figure S4. Thus, the NADPH productivity [as expressed by Eq. (8)] was employed to compare the three strains of the WT, *pgi* mutant, and *ptsG* mutant (Figures 4D–F). In the *pgi* mutant, the NADPH productivity becomes the highest value of 2.9 mmol/l/h at a xylose content of 16% (Figure 4E). This value is the highest among three strains. We have thus established an efficient NADPH production system of the *pgi* mutant on a mixture of glucose and xylose.

### Design of MVA Production Using NADPH Production System of *pgi* Mutant Using a Mixture of Glucose and Xylose

**FIGURE 5:**
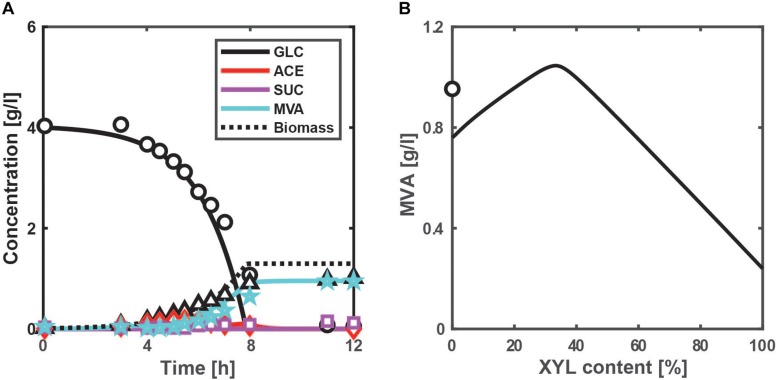
Simulation results of the batch cultivations of the MVA-producing strain under aerobic condition. **(A)** Time-course profile of the extracellular metabolites and biomass concentrations for the case of using glucose, where the lines show simulation results and the symbols represent experimental data (Wada et al., 2017), where an MVA-producing *E. coli* strain was constructed by introducing MVA pathway genes. **(B)** The simulated final MVA concentrations of the *pgi* mutant with the overexpression of ArcA (TF_ArcA_ = 0.95) for the cases of using a mixture of glucose and xylose. The xylose content [%] is defined as the ratio of xylose contained in a mixture. The total substrate concentration is 4 g/l. The symbol shows the experimental data of **(A)** (Wada et al., 2017).

To demonstrate the feasibility of the above NADPH production system, we designed the MVA production system by adding three-step reactions from acetyl-CoA (AcCoA) to the present model (Figure 1), where MVA pathway genes from *Enterococcus faecalis* were incorporated. MVA is an important precursor of value-added chemicals such as isoprenoid. The kinetic model equations and parameter values are given in Supplementary Material. Figure 5A shows the simulated time courses of the extracellular metabolites and biomass concentrations for the batch culture of the MVA-producing strain of *E. coli* grown on glucose under aerobic condition. Acetate production (which comes from AcCoA) reduces as compared to the WT strain (Supplementary Figure S4A), while MVA production increases as experimentally observed (Wada et al., 2017). The correlation coefficient between the simulated and measured intracellular fluxes is 0.976 (*p* < 0.01), as shown in Supplementary Figure S7. These results indicate that our model is effective in designing the MVA production.

In the *pgi* mutant with the MVA-producing pathway, the optimal xylose content shifts from 16% to 5% (more glucose rich side) (Supplementary Figure S8), because NADPH is partially utilized by MVA pathways. Relaxed inhibition of G6PDH by NADPH decreases G6P accumulation, which is predicted to increase the specific GUR. Unexpectedly, the MVA flux in the *pgi* mutant using the optimal xylose content is rather lower than the wild-type strain with the MVA-producing pathway (Supplementary Figure S9).

### Redesign of MVA Production System

To overcome the limitation of the MVA production rate in the *pgi* mutant with MVA-producing pathway, we rationally redesigned the *pgi* mutant. In addition to the NADPH availability for the industrially important metabolic products such as fatty acids, polyhydroxyalkanoates, and isoprenoids, the availability of AcCoA is also very important, because it is the precursor and the starting metabolite for the production of such chemicals including MVA. However, in the *pgi* mutant, the activity of cAMP-Crp becomes high (Figure 2D and Supplementary Figure S4D), activating most of the TCA cycle genes (Shimada et al., 2011). It results in the decrease in AcCoA (Supplementary Figure S9). Therefore, the overproduction of NADPH by the *pgi* mutant may not necessarily improve the MVA production without increasing AcCoA pool size. Note that the overflow metabolism occurs by the imbalance between the glycolytic and the TCA cycle fluxes, producing acetate from AcCoA (Wolfe, 2005). This implies that the repression of the TCA cycle activity contributes to the production of useful AcCoA deriving metabolite.

While aerobic respiration control (Arc) system (or ArcA) play essential roles during oxygen limitation (micro-aerobic and anaerobic conditions), ArcA represses the expression of the TCA cycle genes even under aerobic condition (Basan et al., 2017). Since it is useful to consider the overexpression of ArcA in the *pgi* mutant to increase the AcCoA pool size, we redesigned the *pgi* mutant with the overexpression of ArcA to increase the final MVA concentration with respect to the xylose content in aerobic batch cultures, as shown in Figure 5B. The *arcA* gene was overexpressed in the present model by setting the activity of the ArcA [expressed by Eq. (S30d) in Supplementary Material] (0 < TF_ArcA_ < 1) to 0.95. The CS (entry enzyme of the TCA cycle) flux decreases; the AcCoA concentration increases; the MVA flux increases while keeping the specific NADPH production rate high (Supplementary Figure S10). As rationally predicted, the overexpression of ArcA in the *pgi* mutant increases the MVA production. Thirty-three percentages of xylose content maximize the MVA production and surpass the experimental data (Wada et al., 2017). The redesign strategy achieves the AcCoA overflow with overexpression of ArcA in the *pgi* mutant, and optimizes the xylose content to avoid the accumulation of G6P while keeping a sufficient amount of NADPH. The redesigned strain enhances MVA production by 9.7%, while its increase rate is still marginal. It is probably because the ArcA-suppressed TCA cycle and respiratory chain (Nuo and Cyo) decrease the ATP production. A next challenge is to improve the substrate uptake rate (SUR) (via substrate-level phosphorylation) to increase the ATP production.

In the production of value-added chemicals such as fatty acids, polyhydoxyalkanoates (PHAs) and MVA, AcCoA is a common precursor and NADPH is an essential coenzyme. A question may arise as to which is a limiting factor, AcCoA or NADPH. According to our simulation results, it depends on the ratio of glucose to xylose and the genetic modifications. In the *pgi* mutant with the MVA-producing pathway, the AcCoA level is critically responsible for the case of using optimized xylose content (33%) (since overexpression of ArcA improves the MVA production), while the NADPH level becomes a limiting factor for the case of using only xylose (since NADPH production at the OPP pathway vanishes).

### Model Extension to the Industrially Relevant High Glucose Concentration

In industrial application, a high glucose concentration of more than 100 g/l is widely used. In such a case, acetate is rapidly produced due to overflow metabolism in *E. coli*. It has been reported that a high acetate concentration of more than 5 g/l inhibited the cell growth (Luli and Strohl, 1990). Thus, the equation for the cell growth rate can be modified as:

(10) $$\mu =k_{A\,T\,P}\cdot v_{A\,T\,P}\cdot \frac{k_{A\,C\,E}}{k_{A\,C\,E}+\left[A\,C\,E\right]}$$

We additionally simulated the aerobic batch growth using 100 g/l of initial glucose concentration as shown in Supplementary Figure S11. The simulated acetate and glucose concentrations were consistent with the experimental data at a glucose concentration of more than 20 g/l (Borja et al., 2012). In this way, the present model that incorporates the inhibitory effect of acetate on the cell growth rate is applicable even at high glucose concentration.

The main purpose of computer simulation is to predict the metabolic behaviors based on essential molecular mechanisms and their associated functions of ‘real’ microorganisms. The proposed kinetic model incorporates both the enzyme-level and gene regulatory mechanisms with major TFs such as cAMP-Crp, Cra, and ArcA. Therefore, it leads to an understanding of molecular mechanisms that generate different metabolic behaviors. The important thing is that the mechanism-based model captures the essential characteristics or functions of “real” microorganism. The present computer model allows us to rationally design the cell metabolism for the useful metabolite production.

## Conclusion

In order to rationally design microbial cell factories that efficiently produce useful metabolites, we need to understand the detailed mechanisms by which metabolic and gene regulatory networks regulate complex cellular functions. We have developed a kinetic model to design an efficient NADPH production system by the *pgi*-knockout mutant using a mixture of glucose and xylose, because NADPH is essential for synthesis of useful compounds such as 3HB, MHB, amino acids, fatty acids, and isoprenoids. The *pgi*-knockout mutant enhances NADPH production with serious cell growth suppression, but xylose addition improves its cell growth. There exists the optimal xylose content that maximizes the NADPH productivity.

We used the proposed efficient NADPH production system to design a production system of MVA, a typical value-added chemical. MVA production requires AcCoA as the precursor and NADPH as the reducing power. We implemented ArcA overexpression into the NADPH production system for the enhanced MVA production. The designed mutant is predicted to overflow AcCoA by suppressing the TCA cycle and to produce NADPH without much reducing cell growth.

The present study exemplifies a rational design for the efficient NADPH synthesis toward useful metabolite production such as MVA production. In particular, we get insight into essential dynamic features for the efficient fermentation and predict the different metabolic characteristics. Of course, mathematical modeling should work together with experimental validation.

This study contributes to great advances in computer-aided design (CAD) of metabolic and gene regulatory networks for enhanced useful compound production (Kurata et al., 2003, 2005). Use of CAD is able to repeatedly improve genetic engineering and culture strategy for achieving purposes of interest. CAD accelerates progresses in synthetic biology that develops microbial cell factories and in systems biology that understands mechanisms by which metabolic and gene regulatory networks generate various cellular functions.

## Data Availability Statement

The program is freely available on http://www.cadlive.jp/cadlive_main/Softwares/KineticModel/Ecolimetabolism.html.

## Author Contributions

YM designed the research, constructed the model, performed the simulations, analyzed the data, and wrote the manuscript. HK designed the research and wrote the manuscript. Both authors read and approved the final manuscript.

## Conflict of Interest

The authors declare that the research was conducted in the absence of any commercial or financial relationships that could be construed as a potential conflict of interest.

## Nomenclature

**Primary Metabolic Pathway and Transport System**
ED pathway: Entner–Doudoroff pathway
EI: enzyme I
EIIA: enzyme IIA
HPr: histidine-phosphorylatable protein
PP pathway: pentose phosphate pathway
PTS: phosphotransferase system
TCA cycle: tricarboxylic acid cycle.

**Metabolites**
ACAL: acetaldehyde
AcCoA: acetyl-CoA
CIT: citrate
DHAP: dihydroxy acetone phosphate
E4P: erythrose-4-phosphate
ETH: ethanol
FBP: fructose-1,6-bisphosphate
FOR: formate
F6P: fructose-6-phosphate
FUM: fumarate
G6P: glucose-6-phosphate
GAP: glycelaldehyde-3-phosphate
GLC: glucose
GOX: glyoxylate
ICI: isocitrate
KDPG: 2-keto-3-deoxyphosphogluconate
αKG: α-ketoglutarate
LAC: lactate
MAL: malate
MVA: mevalonate
OAA: oxaloacetate
PEP: phosphoenol pyruvate
6PG: 6-phosphogluconate
6PGL: 6-phosphogluconolactone
PYR: pyruvate
Q: quinone
QH_2_: quinol
R5P: ribose-5-phosphate
RU5P: ribulose-5-phosphate
S7P: sedoheptulose-7-phosphate
SUC: succinate
XYL: xylose
XYLU: xylulose.

**Enzymes**
Ack: acetate kinase
Acs: acetyl coenzyme A synthetase
ADH: alcohol dehydrogenase
ALDH: acetaldehyde dehydrogenase
Cya: adenylate cyclase
Cyd: cytochrome *bd*
Cyo: cytochrome *bo*
CS: citrate synthase
Eda: 2-keto-3-deoxyphosphogluconate aldolase
Edd: 6-phosphogluconate dehydratase
Eno: enolase
Fba: fructose-1,6-bisphosphate aldolase
Fbp: fructose bisphosphatase
Frd: fumarate reductase
Fum: fumarase
G6PDH: glucose-6-phosphate dehydrogenase
GAPDH: glyceraldehyde-3-phosphate dehydrogenase
Glk: glucokinase
ICDH: isocitrate dehydrogenase
Icl: isocitrate lyase
αKGDH: α-ketoglutarate dehydrogenase
LDH: lactate dehydrogenase
MDH: malate dehydrogenase
Mez: malic enzyme
MS: malate synthase
Ndh: NADH dehydrogenase-II
Nuo: NADH dehydrogenase-I
Pck: phosphoenolpyruvate carboxykinase
PDH: pyruvate dehydrogenase
Pfk: phosphofructokinase
Pfl: pyruvate formate lyase
PGDH: 6-phosphogluconate dehydrogenase
Pgi: phosphoglucose isomerase
Pgk: phosphoglucokinase
Pgm: phosphoglucomutase
Ppc: phosphoenolpyruvate carboxylase
Pps: phosphoenolpyruvate synthase
Pta: phosphotransacetylase
Pyk: pyruvate kinase
Rpe: ribulose phosphate epimerase
Rpi: ribose phosphate isomerase
SDH: succinate dehydrogenase
Tal: transaldolase
TktA: transketolase I
TktB: transketolase II
XT: xylose transport
Xyi: xylose isomerase
Xyk: xylulokinase.
