# Supplementary material for: Computer-Aided Rational Design of Efficient NADPH Production System by Escherichia coli pgi Mutant Using a Mixture of Glucose and Xylose
Source: Front Bioeng Biotechnol. 2020 Apr 7;8:277. doi: 10.3389/fbioe.2020.00277 (PMC7154054; doi:10.3389/fbioe.2020.00277)
Supplement: Supplementary file 1 [file Table_1.PDF]

1. Detailed model equations

1.1. Mass balance equations

Referring to Fig. 1 in the main text, the mass balance equations are expressed as follows:

Extracellular state variables

$$\frac{\mathrm{d}[X]}{\mathrm{d}t} = \mu[X] \tag{S1}$$

$$\frac{\mathrm{d}[GLC]}{\mathrm{d}t} = -v_{GLC,uptake} \tag{S2a}$$

$$\frac{\mathrm{d}[XYL]}{\mathrm{d}t} = -v_{XYL,uptake} \tag{S2b}$$

$$\frac{\mathrm{d}[ACT]}{\mathrm{d}t} = v_{ACT,excrete} - v_{ACT,uptake} \tag{S3a}$$

$$\frac{\mathrm{d}[LAC]}{\mathrm{d}t} = v_{LAC,excrete} \tag{S3b}$$

$$\frac{\mathrm{d}[FOR]}{\mathrm{d}t} = v_{FOR,excrete} \tag{S3c}$$

$$\frac{\mathrm{d}[ETH]}{\mathrm{d}t} = v_{ETH,excrete} \tag{S3d}$$

$$\frac{\mathrm{d}[SUC]}{\mathrm{d}t} = v_{SUC,excrete} \tag{S3e}$$

Intracellular metabolites

$$\frac{\mathrm{d}[GLC^{in}]}{\mathrm{d}t} = v_{NPTS} - v_{Glk} - \mu[GLC^{in}] \tag{S4a}$$

$$\frac{\mathrm{d}[G6P]}{\mathrm{d}t} = v_{PTS4} + v_{Glk} - v_{Pgi} - v_{G6PDH} - v_{Bio,G6P} - \mu[G6P] \tag{S4b}$$

$$\frac{\mathrm{d}[F6P]}{\mathrm{d}t} = v_{Pgi} + v_{Fbp} + v_{TktB} + v_{Tal} - v_{Pfk} - v_{Bio,F6P} - \mu[F6P] \tag{S4c}$$

$$\frac{\mathrm{d}[FBP]}{\mathrm{d}t} = v_{Pfk} - v_{Fba} - v_{Fbp} - \mu[FBP] \tag{S4d}$$

$$\begin{aligned}\frac{\mathrm{d}[GAP/DHAP]}{\mathrm{d}t} &= 2v_{Fba} + v_{TktA} + v_{TktB} + v_{Eda} \\ &\quad - v_{L\_Emp} - v_{Tal} - v_{Bio,GAP} - \mu[GAP/DHAP]\end{aligned} \tag{S4e}$$

$$\frac{\mathrm{d}[PEP]}{\mathrm{d}t} = v_{L\_Emp} + v_{Pck} + v_{Pps} - v_{Pyk} - v_{Ppc} - v_{PTS1} - v_{Bio,PEP} - \mu[PEP] \tag{S4f}$$

$$\frac{\mathrm{d}[PYR]}{\mathrm{d}t} = v_{Pyk} + v_{Mez} + v_{PTS1} + v_{Eda} - v_{PDH} - v_{Pps} - v_{LDH} - v_{Pfl} - v_{Bio,PYR} - \mu[PYR] \tag{S4g}$$

$$\begin{aligned}\frac{\mathrm{d}[AcCoA]}{\mathrm{d}t} &= v_{PDH} + v_{Acs} + v_{Pfl} \\ &\quad - v_{CS} - v_{PTACK} - v_{MS} - v_{ALDH} - v_{Bio,AcCoA} - \mu[AcCoA]\end{aligned} \tag{S4h}$$

$$\frac{\mathrm{d}[ACAL]}{\mathrm{d}t} = v_{ALDH} - v_{ADH} - \mu[ACAL] \tag{S4i}$$

$$\frac{\mathrm{d}[PGL/6PG]}{\mathrm{d}t} = v_{G6PDH} - v_{PGDH} - v_{Edd} - \mu[PGL/6PG] \tag{S4j}$$

$$\frac{\mathrm{d}[RU5P]}{\mathrm{d}t} = v_{PGDH} - v_{Rpe} - v_{Rpi} - \mu[RU5P] \tag{S4k}$$

$$\frac{\mathrm{d}[R5P]}{\mathrm{d}t} = v_{Rpi} - v_{TktA} - v_{Bio,R5P} - \mu[R5P] \tag{S4l}$$

$$\frac{\mathrm{d}[X5P]}{\mathrm{d}t} = v_{Rpe} + v_{Xyk} - v_{TktA} - v_{TktB} - \mu[X5P] \tag{S4m}$$

$$\frac{\mathrm{d}[S7P]}{\mathrm{d}t} = v_{TktA} - v_{Tal} - \mu[S7P] \tag{S4n}$$

$$\frac{\mathrm{d}[E4P]}{\mathrm{d}t} = v_{Tal} - v_{TktB} - v_{Bio,E4P} - \mu[E4P] \tag{S4o}$$

$$\frac{\mathrm{d}[XYL^{in}]}{\mathrm{d}t} = v_{XT} - v_{Xyi} - \mu[XYL^{in}] \tag{S4p}$$

$$\frac{\mathrm{d}[XYLU]}{\mathrm{d}t} = v_{Xyi} - v_{Xyk} - \mu[XYLU] \tag{S4q}$$

$$\frac{\mathrm{d}[KDPG]}{\mathrm{d}t} = v_{Edd} - v_{Eda} - \mu[S7P] \tag{S4r}$$

$$\frac{\mathrm{d}[CIT/ICIT]}{\mathrm{d}t} = v_{CS} - v_{ICDH} - v_{Icl} - \mu[CIT/ICIT] \tag{S4s}$$

$$\frac{\mathrm{d}[\alpha KG]}{\mathrm{d}t} = v_{ICDH} - v_{\alpha KGDH} - v_{Bio,\alpha KG} - \mu[\alpha KG] \tag{S4t}$$

$$\frac{\mathrm{d}[SUC^{in}]}{\mathrm{d}t} = v_{\alpha KGDH} + v_{Icl} + v_{Frd} - v_{SDH} - v_{SUC_{trans}} - \mu[SUC^{in}] \tag{S4u}$$

$$\frac{\mathrm{d}[MAL]}{\mathrm{d}t} = v_{SDH} + v_{MS} - v_{MDH} - v_{Frd} - v_{Mez} - \mu[MAL] \tag{S4v}$$

$$\frac{\mathrm{d}[OAA]}{\mathrm{d}t} = v_{MDH} + v_{Ppc} - v_{CS} - v_{Pck} - v_{Bio,OAA} - \mu[OAA] \quad \text{(S4w)}$$

$$\frac{\mathrm{d}[GOX]}{\mathrm{d}t} = v_{Icl} - v_{MS} - \mu[GOX] \quad \text{(S4x)}$$

PTS proteins, cAMP, and transcription factors

$$\frac{\mathrm{d}[EIIA]}{\mathrm{d}t} = v_{PTS4} - v_{PTS1} \quad \text{(S5a)}$$

$$\frac{\mathrm{d}[EIIA \sim P]}{\mathrm{d}t} = v_{PTS1} - v_{PTS4} \quad \text{(S5b)}$$

$$\frac{\mathrm{d}[cAMP]}{\mathrm{d}t} = v_{Cya} - v_{cAMP\,\mathrm{deg}r} - \mu[cAMP] \quad \text{(S5c)}$$

$$\frac{\mathrm{d}[cAMP - Crp]}{\mathrm{d}t} = v_{cAMP-Crp} - \mu[cAMP - Crp] \quad \text{(S6a)}$$

$$\frac{\mathrm{d}[Cra - FBP]}{\mathrm{d}t} = v_{Cra-FBP} - \mu[Cra - FBP] \quad \text{(S6b)}$$

$$\frac{\mathrm{d}[PdhR - PYR]}{\mathrm{d}t} = v_{PdhR-PYR} - \mu[PdhR - PYR] \quad \text{(S6c)}$$

Quinone (Q)/quinol (QH_2) redox couple and NADH

$$\frac{\mathrm{d}[QH_2]}{\mathrm{d}t} = v_{Nuo} + v_{Ndh} + v_{Syn,QH_2} + v_{SDH} - v_{Cyo} - v_{Cyd} - \mu[QH_2] \quad \text{(S7a)}$$

$$\frac{\mathrm{d}[Q]}{\mathrm{d}t} = v_{Cyo} + v_{Cyd} - v_{Nuo} - v_{Ndh} - v_{SDH} - \mu[Q] \quad \text{(S7b)}$$

$$\frac{\mathrm{d}[NADH]}{\mathrm{d}t} = v_{Emp} + v_{PDH} + v_{\alpha KGDH} + v_{MDH} + v_{SfcA} - v_{LDH} - v_{ALDH} - v_{ADH} - v_{Nuo} - v_{Ndh} - \mu[NADH] \quad \text{(S8a)}$$

$$\frac{\mathrm{d}[NADPH]}{\mathrm{d}t} = v_{G6PDH} + v_{PGDH} + v_{ICDH} + v_{MaeB} - v_{Anabolic,NADPH} - \mu[NADPH] \quad \text{(S8b)}$$

where μ is the specific growth rate, and $[X]$ is the biomass concentration. The suffix "Bio" of $v_{Bio,\cdot}$ means the biosynthetic pathway. $v_{\bullet}$ is the function of substrate(s), product(s), or metabolite(s) which allosterically affect the reaction rate as well as the function of transcription factors based on the relationship as given in Fig. 1 in the main text. Moreover, the total concentrations for transcription factor and NADH

are expressed as follows:

$$[Crp]_{total} = [Crp] + [cAMP - Crp] \quad \text{(S9a)}$$

$$[Cra]_{total} = [Cra] + [Cra - FBP] \quad \text{(S9b)}$$

$$[PdhR]_{total} = [PdhR] + [PdhR - PYR] \quad \text{(S9c)}$$

$$[NA*]_{total} = [NADH] + [NAD] \quad \text{(S9d)}$$

1.2. Kinetic model equations

1.2.1. Modeling for PTS

For the glucose phosphorylation step of PTS, a reversible ping-pong mechanism and second-order rate law was considered (Kremling et al., 2001). Here, we assumed EI and HPr to be in equilibrium, and considered only EIIA and phosphorylated EIIA, EIIA-P in relation to PEP, PYR, Glc and G6P (Eqs. S5a and S5b). The resulting v_{PTS1} and v_{PTS4} may be expressed as (Kotte et al., 2010)

$$v_{PTS1} = k_1[PEP][EIIA] - k_{-1}[PYR][EIIA \sim P] \quad \text{(S11a)}$$

$$v_{PTS4} = \frac{v_{PTS4}^{\max}\left[EIIA \sim P\right][Glc]}{\left(K_{EIIA\sim P} + \left[EIIA \sim P\right]\right)\left(K_{Glc} + \left[Glc\right]\right)} \quad \text{(S11b)}$$

We further modified the above equation by considering the effect of the accumulation of the phosphate sugar such as G6P on the destabilization of the *ptsG* mRNA (Morita et al., 2003) as follows

$$v_{PTS4} = v_{PTS4}^{\max} \cdot \frac{{K_{G6P}}^{n}}{{K_{G6P}}^{n} + G6P^{n}} \cdot \frac{\left[EIIA \sim P\right][Glc]}{\left(K_{EIIA\sim P} + \left[EIIA \sim P\right]\right)\left(K_{Glc} + \left[Glc\right]\right)} \quad \text{(S11c)}$$

1.2.2. Modeling for glucose uptake by non-PTS pathway

The glucose uptake via non-PTS transporters such as MglBAC and GalP is followed by

the phosphorylation by Glk. Here, all of these transport systems other than glucose-PTS were assumed to be represented by NPTS, and v_{NPTS} and v_{Glk} were expressed as (Bettenbrock et al., 2006)

$$v_{NPTS} = \frac{v_{NPTS}^{\max}[GLC]}{K_S + \left(1 + \frac{[EIIA]}{K_I}\right)[GLC]} \tag{S12a}$$

$$v_{Glk} = \frac{v_{Glk}^{\max}[GLC^{in}]}{\left(1 + \frac{[G6P]}{K_I}\right)\left(K_S + [GLC^{in}]\right)} \tag{S12b}$$

1.2.3. Modeling for cAMP

Cya (adenylate cyclase) generates cAMP, and its transcription is activated by EIIA-P. The cAMP generation (v_{Cya}) and degradation ($v_{cAMP\deg r}$) were expressed as (Kremling et al., 2001; Kotte et al., 2010)

$$v_{Cya} = \frac{v_{Cya}^{\max}[EIIA \sim P]}{[EIIA \sim P] + K_{EIIA\sim P}} \tag{S13a}$$

$$v_{cAMP\deg r} = \frac{v_{Degr}^{\max}[cAMP]}{[cAMP] + K_{cAMP}} \tag{S13b}$$

1.2.4. Modeling for glycolysis

The kinetic model equations have been proposed by several researchers in the past (Chassagnole et al., 2002; Kotte et al., 2010). Here, the reaction rate for Pgi was expressed as (Chassagnole et al., 2002)

$$v_{Pgi} = \frac{v_{Pgi}^{\max}\left([G6P] - \frac{[F6P]}{K_{eq}}\right)}{K_{G6P}\left(1 + \frac{[F6P]}{K_{F6P}\left(1 + \frac{[6PG]}{K_{F6P,6PGinh}}\right)} + \frac{[6PG]}{K_{G6P,6PGinh}}\right) + [G6P]} \tag{S14a}$$

The reaction rate for Pfk was expressed as a function of its substrate F6P and allosteric inhibition by PEP such as (Kotte et al., 2010)

$$v_{Pfk} = \frac{v_{Pfk}^{\max}\frac{[F6P]}{K_{F6P}}\left(1 + \frac{[F6P]}{K_{F6P}}\right)^{n-1}}{\left(1 + \frac{[F6P]}{K_{F6P}}\right)^{n} + L\left(1 + \frac{[PEP]}{K_{PEP}}\right)^{n}} \tag{S14b}$$

The reaction rate through Fba was expressed as (Kotte et al., 2010)

$$v_{Fba} = \frac{v_{Fba}^{\max,f}\frac{[FBP]}{K_{FBP}} - v_{Fba}^{\max,r}\frac{[GAP/DHAP]}{K_{GAP/DHAP}}}{1 + \frac{[FBP]}{K_{FBP}} + \frac{[GAP/DHAP]}{K_{GAP/DHAP}}} \tag{S14c}$$

Then, the reactions through GAPDH, Pgk, Pgm, and Eno were lumped together as (Kotte et al., 2010)

$$v_{L\_Emp} = \frac{v_{L\_Emp}^{\max,f}\frac{[GAP/DHAP]}{K_{GAP/DHAP}} - v_{L\_Emp}^{\max,r}\frac{[PEP]}{K_{PEP}}}{1 + \frac{[GAP/DHAP]}{K_{GAP/DHAP}} + \frac{[PEP]}{K_{PEP}}} \tag{S14d}$$

The reaction rate for Pyk was expressed as (Kotte et al., 2010)

$$v_{Pyk} = \frac{v_{Pyk}^{\max}\frac{[PEP]}{K_{PEP}}\left(1 + \frac{[PEP]}{K_{PEP}}\right)^{n-1}}{\left(1 + \frac{[PEP]}{K_{PEP}}\right)^{n} + L\left(1 + \frac{[FBP]}{K_{FBP}}\right)^{-n}} \tag{S14e}$$

where this is a function of its substrate PEP and the allosteric activator FBP.

1.2.5. Modeling for PP pathways

The kinetic model equations have also been proposed by several researchers (Chassagnole et al., 2002). Here oxidative PP pathway reactions such as v_{G6PDH} and v_{PGDH} were expressed as a growth rate dependent reaction (Wolf et al. 1979) based on the similar equations as (Chassagnole et al., 2002)

$$v_{G6PDH} = \frac{v_{G6PDH}^{\max}[G6P][NADP]}{\left([G6P]+K_{G6P}\right)\left(1+\frac{[NADPH]}{K_{NADPH\_G6Pinh}}\right)\left(K_{NADP}\left(1+\frac{[NADPH]}{K_{NADPH\_NADPinh}}\right)+[NADP]\right)} \quad \text{(S15a)}$$

$$v_{PGDH} = \frac{v_{PGDH}^{\max}[6PG][NADP]}{\left([6PG]+K_{6PG}\right)\left([NADP]+K_{NADP}\left(1+\frac{[NADPH]}{K_{NADPHinh}}\right)\left(1+\frac{[ATP]}{K_{ATPinh}}\right)\right)} \quad \text{(S15b)}$$

where $v_{G6PDH}^{\max} = \left(k_{G6PDH}\cdot\mu + k_{G6PDH}^{'}\right)v_{G6PDH}^{\max *}$ and $v_{PGDH}^{\max} = \left(k_{PGDH}\cdot\mu + k_{PGDH}^{'}\right)v_{PGDH}^{\max *}$. The values of k_{G6PDH}, $k_{G6PDH}^{'}$, k_{PGDH}, and $k_{PGDH}^{'}$ were obtained from the experimental data by assuming linear relationship between the relative gene expression (RGE) and the dilution rate or the specific growth rate (Yao et al., 2011). Specifically, the following equation was assumed for gene expression of G6PDH.

$$RGE_{G6PDH} = k_{G6PDH}\cdot\mu + k_{G6PDH}^{'} \quad \text{(S15c)}$$

The maximum velocity of G6PDH is expressed as

$$v_{G6PDH}^{\max} = k_{cat}\cdot[G6PDH] \quad \text{(S15d)}$$

Dividing this equation by enzyme concentration of house-keeping gene ($[E]*$), we have

$$v_{G6PDH}^{\max}/[E]* = k_{cat}\cdot[G6PDH]/[E]* \quad \text{(S15e)}$$

Given that $[G6PDH]/[E]* \approx RGE_{G6PDH}$, we can derive the equation that $v_{G6PDH}^{\max}$ equals to $\left(k_{G6PDH}\cdot\mu + k_{G6PDH}^{'}\right)v_{G6PDH}^{\max *}$, where $v_{G6PDH}^{\max *} = k_{cat}\cdot[E]*$. In the same manner,

$v_{PGDH}^{\max}$ of PGDH was also derived.

Non-oxidative PP pathway reactions were expressed as (Chassagnole et al., 2002)

$$v_{Rpe} = v_{Rpe}^{\max}\left([RU5P] - \frac{[X5P]}{K_{eq}}\right) \tag{S16a}$$

$$v_{Rpi} = v_{Rpi}^{\max}\left([RU5P] - \frac{[R5P]}{K_{eq}}\right) \tag{S16b}$$

$$v_{TktA} = v_{TktA}^{\max}\left([R5P][X5P] - \frac{[S7P][GAP/DHAP]}{K_{eq}}\right) \tag{S16c}$$

$$v_{TktB} = v_{TktB}^{\max}\left([X5P][E4P] - \frac{[F6P][GAP/DHAP]}{K_{eq}}\right) \tag{S16d}$$

$$v_{Tal} = v_{Tal}^{\max}\left([GAP/DHAP][S7P] - \frac{[E4P][F6P]}{K_{eq}}\right) \tag{S16e}$$

1.2.6. Modeling for ED pathways

The equations for Eda and Edd were expressed as follows (Peskov et al. 2012):

$$v_{Edd} = \frac{v_{Edd}^{\max} Q_{pH}\left([6PG] - \frac{[KDPG]}{K_{eq}}\right)}{K_{6PG} + [6PG] + \frac{K_{6PG}}{K_{KDPG}}[KDPG]} \tag{S17a}$$

$$Q_{pH} = \frac{1 + 2\cdot 10^{(pH_m - pK)}}{1 + 10^{(pH - pK)} + 10^{(2\cdot pH_m - pH - pK)}}$$

$$v_{Eda} = \frac{v_{Edd}^{\max} Q_{pH}\left([KDPG] - \frac{[GAP/DHAP][PYR]}{K_{eq}}\right)}{K_{KDPG} + [KDPG] + K_{KDPG}\left(\frac{[PYR]}{K_{PYR}} + \frac{[GAP/DHAP]}{K_{GAP/DHAP}} + \frac{[PYR][GAP/DHAP]}{K_{PYR}K_{GAP/DHAP}}\right)} \tag{S17b}$$

$$Q_{pH} = \frac{1 + 2\cdot 10^{(pH_m - pK)}}{1 + 10^{(pH - pK)} + 10^{(2\cdot pH_m - pH - pK)}}$$

1.2.7. Modeling for fermentative pathways

The lactate is formed by LDH, and the rate equation was expressed as a function of two substrates, PYR and NADH. The Hill equation was used based on the experimental observation (Dearriaga et al. 1982) as follows:

$$v_{LDH} = v_{LDH}^{\max} \cdot \frac{[NADH]^{n_{NADH}}}{K_{NADH}{}^{n_{NADH}} + [NADH]^{n_{NADH}}} \cdot \frac{[PYR]^{n_{PYR}}}{K_{PYR}{}^{n_{PYR}} + [PYR]^{n_{PYR}}} \tag{S18a}$$

The formate is formed by Pfl, and its reaction was expressed as (Cintolesi et al. 2012)

$$v_{Pfl} = \frac{v_{Pfl}^{\max,f}[PYR][CoA]}{[PYR][CoA] + K_{PYR}[CoA] + K_{CoA}[PYR]} - \frac{v_{Pfl}^{\max,r}[AcCoA][FOR]}{[AcCoA][FOR] + K_{AcCoA}[FOR] + K_{FOR}[AcCoA]} \tag{S18b}$$

The ethanol is formed by ADH. ALDH is used to convert AcCoA into acetaldehyde (AcAld), and the subsequent reduction of AcAld produces ethanol. These rate equations were expressed as (Hoefnagel et al. 2002)

$$v_{ALDH} = \frac{v_{ALDH}^{\max}\left([AcCoA][NADH] - \frac{[CoA][NAD][ACAL]}{K_{eq}}\right)}{\left(K_{AcCoA}K_{NADH} + \frac{K_{AcCoA}K_{NADH}[NAD]}{K_{NAD}} + K_{AcCoA}[NADH]\right)\left(1 + \frac{[AcCoA]}{K_{AcCoA}} + \frac{[CoA]}{K_{CoA}} + \frac{[ACAL]}{K_{ACAL}} + \frac{[ACAL][CoA]}{K_{ACAL}K_{CoA}}\right)} \tag{S18c}$$

$$v_{ADH} = \frac{v_{ADH}^{\max}\left([ACAL][NADH] - \frac{[ETH][NAD]}{K_{eq}}\right)}{\left(K_{ACAL}K_{NADH} + \frac{K_{ACAL}K_{NADH}[NAD]}{K_{NAD}} + K_{ACAL}[NADH]\right)\left(1 + \frac{[ACAL]}{K_{ACAL}} + \frac{[ETH]}{K_{ETH}}\right)} \tag{S18d}$$

1.2.8. Modeling for acetate formation

Acetate is formed from AcCoA by the reactions of Pta and Ack, and here we lumped these reactions together as (Kotte et al. 2010)

$$v_{PTACK} = \frac{v_{PTACK}^{\max}\frac{[AcCoA]}{K_{AcCoA}}\left(1 + \frac{[AcCoA]}{K_{AcCoA}}\right)^{n-1}}{\left(1 + \frac{[AcCoA]}{K_{AcCoA}}\right)^{n} + L\left(1 + \frac{[PYR]}{K_{PYR}}\right)^{-n}} \tag{S19a}$$

Acetate consumption rate was expressed as (Kotte et al. 2010)

$$v_{Acs} = \frac{v_{Acs}^{\max}[ACT]}{[ACT] + K_{ACT}} \tag{S19b}$$

1.2.9. Modeling for PDH and TCA cycle

The equation for PDH was expressed as (Kotte et al. 2010)

$$v_{PDH} = \frac{v_{PDH}^{\max} \frac{[PYR]}{K_{PYR}} \left(1 + \frac{[PYR]}{K_{PYR}}\right)^{n-1}}{\left(1 + \frac{[PYR]}{K_{PYR}}\right)^{n} + L\left(1 + \frac{[GOX]}{K_{GOX}} + \frac{[PYR]}{K_{I,PYR}}\right)^{n}} \tag{S20a}$$

The equation for CS was expressed as (Kotte et al. 2010)

$$v_{CS} = \frac{v_{CS}^{\max}[AcCoA][OAA]}{\left(1 + \frac{[\alpha KG]}{K_{\alpha KG}}\right)K_{OAA,AcCoA}K_{AcCoA} + K_{AcCoA}[OAA] + \left(1 + \frac{[\alpha KG]}{K_{\alpha KG}}\right)K_{OAA}[AcCoA] + [AcCoA][OAA]} \tag{S20b}$$

The rate equation for ICDH was expressed as (Kotte et al. 2010)

$$v_{ICDH} = \frac{v_{ICDH}^{\max} \frac{[CIT/ICIT]}{K_{CIT/ICIT}} \left(1 + \frac{[CIT/ICIT]}{K_{CIT/ICIT}}\right)^{n-1}}{\left(1 + \frac{[CIT/ICIT]}{K_{CIT/ICIT}}\right)^{n} + L\left(1 + \frac{[PEP]}{K_{PEP}}\right)^{n}} \tag{S20c}$$

The ICDH loses the activity via phosphorylation by ICDH kinase which is inhibited by OAA. We, therefore, modified the above equation by considering the effect of OAA concentration on the activity of ICDH as follows:

$$v_{ICDH} = v_{ICDH}^{\max} \cdot \frac{[OAA]}{[OAA] + K_{OAA}} \cdot \frac{\frac{[CIT/ICIT]}{K_{CIT/ICIT}} \left(1 + \frac{[CIT/ICIT]}{K_{CIT/ICIT}}\right)^{n-1}}{\left(1 + \frac{[CIT/ICIT]}{K_{CIT/ICIT}}\right)^{n} + L\left(1 + \frac{[PEP]}{K_{PEP}}\right)^{n}} \tag{S20d}$$

The equation for αKGDH was expressed as (Kotte et al. 2010)

$$v_{\alpha KGDH} = \frac{v_{\alpha KGDH}^{\max}[\alpha KG]}{[\alpha KG] + K_{\alpha KG}} \tag{S20e}$$

Here, the forward and backward fluxes of SDH/Frd reactions used by Usuda et al. (2010) were considered as SDH and Frd reactions, respectively.

$$v_{SDH} = \frac{v_{SDH}^{\max}[SUC^{in}]}{K_{SUC} + [SUC^{in}] + \frac{K_{SUC}[FUM/MAL]}{K_{FUM/MAL}}} \tag{S20f}$$

$$v_{Frd} = \frac{v_{Frd}^{\max}[FUM/MAL]}{K_{FUM/MAL} + [FUM/MAL] + \frac{K_{FUM/MAL}[SUC^{in}]}{K_{SUC}}} \tag{S20g}$$

The MDH reaction was expressed as (Usuda et al. 2010)

$$v_{MDH} = \frac{v_{MDH}^{\max,f} K_{OAA} K_{NADH}[FUM/MAL][NAD] - v_{MDH}^{\max,r} K_{FUM/MAL} K_{NAD}[OAA][NADH]}{\left(K_{FUM/MAL}K_{NAD} + K_{NAD}[FUM/MAL] + \frac{K_{FUM/MAL}K_{NAD}[OAA]}{K_{OAA}}\right)\left(K_{OAA}K_{NADH} + K_{OAA}[NADH] + \frac{K_{OAA}K_{NADH}[NAD]}{K_{NAD}}\right)} \tag{S20h}$$

1.2.10. Modeling for glyoxylate pathways

The rate equations for glyoxylate pathway were expressed as (Kotte et al. 2010)

$$v_{Icl} = \frac{v_{Icl}^{\max} \frac{[CIT/ICIT]}{K_{CIT/ICIT}} \left(1 + \frac{[CIT/ICIT]}{K_{CIT/ICIT}}\right)^{n-1}}{\left(1 + \frac{[CIT/ICIT]}{K_{CIT/ICIT}}\right)^{n} + L\left(1 + \frac{[PEP]}{K_{PEP}} + \frac{[GAP/\sim/2PG]}{K_{GAP/\sim/2PG}} + \frac{[\alpha KG]}{K_{\alpha KG}}\right)^{n}} \tag{S21a}$$

$$v_{MS} = \frac{v_{MS}^{\max}[GOX][AcCoA]}{K_{GOXAcCoA}K_{AcCoA} + K_{AcCoA}[GOX] + K_{GOX}[AcCoA] + [GOX][AcCoA]} \tag{S21b}$$

1.2.11. Modeling for gluconeogenesis

The equations for Fbp, Pps, and Pck were expressed as (Kotte et al. 2010)

$$v_{Fbp} = \frac{v_{Fbp}^{\max} \frac{[FBP]}{K_{FBP}} \left(1 + \frac{[FBP]}{K_{FBP}}\right)^{n-1}}{\left(1 + \frac{[FBP]}{K_{FBP}}\right)^{n} + L\left(1 + \frac{[PEP]}{K_{PEP}}\right)^{-n}} \tag{S22a}$$

$$v_{Pps} = \frac{v_{Pps}^{\max} \frac{[PYR]}{K_{PYR}} \left(1 + \frac{[PYR]}{K_{PYR}}\right)^{n-1}}{\left(1 + \frac{[PYR]}{K_{PYR}}\right)^{n} + L\left(1 + \frac{[PEP]}{K_{PEP}}\right)^{n}} \tag{S22b}$$

$$v_{Pck} = \frac{v_{Pck}^{\max}[OAA]}{[OAA] + K_{OAA}\left(1 + \frac{[PEP]}{K_{PEP}}\right)} \tag{S22c}$$

Mez reaction is catalyzed by two enzyme reactions, such as MaeB and SfcA in which each requires $NADP^+$ or NAD^+ as a cofactor, respectively. The equation for MaeB and SfcA were expressed as (Usuda et al., 2010)

$$v_{MaeB} = \frac{v_{MaeB}^{\max}[MAL]^n[NADP^+]}{\left(K_{MAL}{}^n + [MAL]^n\right)\left(K_{NADP^+} + [NADP^+]\right)} \tag{S22d}$$

$$v_{SfcA} = \frac{v_{SfcA}^{\max}[MAL]^n[NAD^+]}{\left(K_{MAL}{}^n + [MAL]^n\right)\left(K_{NAD^+} + [NAD^+]\right)} \tag{S22e}$$

1.2.12. Modeling for anaplerotic reaction

The rate equation for Ppc was expressed as (Kotte et al. 2010)

$$v_{Ppc} = \frac{v_{Ppc}^{\max}\frac{[PEP]}{K_{PEP}}\left(1+\frac{[PEP]}{K_{PEP}}\right)^{n-1}}{\left(1+\frac{[PEP]}{K_{PEP}}\right)^{n} + L\left(1+\frac{[FBP]}{K_{FBP}}\right)^{-n}} \tag{S23a}$$

In addition, AcCoA is also an activator of Ppc reaction, and therefore, we modified the above equation as follows:

$$v_{Ppc} = \frac{v_{Ppc}^{\max}\frac{[PEP]}{K_{PEP}}\left(1+\frac{[PEP]}{K_{PEP}}\right)^{n-1}}{\left(1+\frac{[PEP]}{K_{PEP}}\right)^{n} + L\left(1+\frac{[FBP]}{K_{FBP}}+\frac{[AcCoA]}{K_{AcCoA}}\right)^{-n}} \tag{S23b}$$

1.2.13. Modeling for respiratory chain

The rate reactions of Nuo and Ndh were expressed as (Henkel et al. 2014)

$$v_{Nuo} = v_{Nuo}^{\max}\cdot\frac{[Q]}{K_{m,Nuo,Q}+[Q]}\cdot\frac{[NADH]}{K_{m,Nuo,NADH}+[NADH]} \tag{S24a}$$

$$v_{Ndh} = v_{Ndh}^{\max} \cdot \frac{[Q]}{K_{m,Ndh,Q} + [Q]} \cdot \frac{[NADH]}{K_{m,Ndh,NADH} + [NADH]} \quad \text{(S24b)}$$

The rate reactions of Cyo and Cyd were expressed as (Henkel et al. 2014)

$$v_{Cyo} = v_{Cyo}^{\max} \cdot \frac{[O_2]}{K_{m,Cyo,O_2} + [O_2]} \cdot \frac{[QH_2]}{K_{m,Cyo,QH_2} + [QH_2]} \quad \text{(S24c)}$$

$$v_{Cyd} = v_{Cyd}^{\max} \cdot \frac{[O_2]}{K_{m,Cyd,O_2} + [O_2]} \cdot \frac{[QH_2]}{K_{m,Cyd,QH_2} + [QH_2]} \quad \text{(S24d)}$$

The quinol synthetic rate was expressed based on Henkel et al. (2014) as follows:

$$v_{Syn,QH_2} = \mu \cdot p_{syn,QH_2} \quad \text{(S24e)}$$

where it is produced by biosynthesis, and therefore μ-dependent.

1.2.14. Modeling for SUC transport

SUC transport was expressed as

$$v_{SUC_{trans}} = \frac{v_{SUC_{trans}}^{\max}[SUC^{in}]}{K_{SUC} + [SUC^{in}]} \quad \text{(S25)}$$

1.2.15. Modeling for biosynthetic pathway

The biosynthetic fluxes were expressed as growth dependent as follows:

$$v_{Bio,G6P} = \left(k_{Bio,G6P}^{+} \cdot \mu + k_{Bio,G6P}^{++}\right)[G6P] \quad \text{(S26a)}$$

$$v_{Bio,F6P} = \left(k_{Bio,F6P}^{+} \cdot \mu + k_{Bio,F6P}^{++}\right)[F6P] \quad \text{(S26b)}$$

$$v_{Bio,GAP/DHAP} = \left(k_{Bio,GAP/DHAP}^{+} \cdot \mu + k_{Bio,GAP/DHAP}^{++}\right)[GAP/DHAP] \quad \text{(S26c)}$$

$$v_{Bio,PEP} = \left(k_{Bio,PEP}^{+} \cdot \mu + k_{Bio,PEP}^{++}\right)[PEP] \quad \text{(S26d)}$$

$$v_{Bio,PYR} = \left(k_{Bio,PYR}^{+} \cdot \mu + k_{Bio,PYR}^{++}\right)[PYR] \quad \text{(S26e)}$$

$$v_{Bio,AcCoA} = \left(k^{+}_{Bio,AcCoA} \cdot \mu + k^{++}_{Bio,AcCoA}\right)[AcCoA] \quad \text{(S26f)}$$

$$v_{Bio,R5P} = \left(k^{+}_{Bio,R5P} \cdot \mu + k^{++}_{Bio,R5P}\right)[R5P] \quad \text{(S26g)}$$

$$v_{Bio,E4P} = \left(k^{+}_{Bio,E4P} \cdot \mu + k^{++}_{Bio,E4P}\right)[E4P] \quad \text{(S26h)}$$

$$v_{Bio,\alpha KG} = \left(k^{+}_{Bio,\alpha KG} \cdot \mu + k^{++}_{Bio,\alpha KG}\right)[\alpha KG] \quad \text{(S26i)}$$

$$v_{Bio,OAA} = \left(k^{+}_{Bio,OAA} \cdot \mu + k^{++}_{Bio,OAA}\right)[OAA] \quad \text{(S26j)}$$

where the value of $k^{++}_{Bio,\bullet}$ is several order lower than that of $k^{+}_{Bio,\bullet}$, and therefore, $k^{++}_{Bio,\bullet}$ can be omitted in practice. Moreover, the anabolic flux of NADPH drain can be expressed as follows (Christodoulou et al., 2018):

$$v_{Anabolic} = k_{NADPH} \cdot [NADPH] \quad \text{(S26k)}$$

1.2.16. Modeling for transcription factors

For the interactions of the transcription factors, following equations were used (Kotte et al., 2010)

$$v_{cAMP-Crp} = p_{cAMP-Crp}\left\{\frac{\left([Crp]+[cAMP-Crp]\right)[cAMP]^{n}}{[cAMP]^{n} + K_{cAMP}{}^{n}} - [cAMP-Crp]\right\} \quad \text{(S27a)}$$

$$v_{Cra-FBP} = p_{Cra-FBP}\left\{\frac{\left([Cra]+[Cra-FBP]\right)[FBP]^{n}}{[FBP]^{n} + K_{FBP}{}^{n}} - [Cra-FBP]\right\} \quad \text{(S27b)}$$

$$v_{PdhR-PYR} = p_{PdhR-PYR}\left\{\frac{\left([PdhR]+[PdhR-PYR]\right)[PYR]^{n}}{[PYR]^{n} + K_{PYR}{}^{n}} - [PdhR-PYR]\right\} \quad \text{(S27c)}$$

where $p_{\bullet}$ is the growth-independent model parameter.

1.2.17. Modeling for extracellular metabolite uptake/excretion rates

The extracellular metabolite uptake/excretion rates were expressed by the following equations:

$$v_{GLC,uptake} = M_w^{GLC} \cdot [X^*] \cdot \left(v_{PTS4} + v_{NPTS}\right) \tag{S28a}$$

$$v_{XYL,uptake} = M_w^{XYL} \cdot [X^*] \cdot v_{XT} \tag{S28b}$$

$$v_{ACT,excrete} = M_w^{ACT} \cdot [X^*] \cdot v_{PTACK} \tag{S28c}$$

$$v_{ACT,uptake} = M_w^{ACT} \cdot [X^*] \cdot v_{Acs} \tag{S28d}$$

$$v_{LAC,excrete} = M_w^{LAC} \cdot [X^*] \cdot v_{LDH} \tag{S28e}$$

$$v_{FOR,excrete} = M_w^{FOR} \cdot [X^*] \cdot v_{Pfl} \tag{S28f}$$

$$v_{ETH,excrete} = M_w^{ETH} \cdot [X^*] \cdot v_{ADH} \tag{S28g}$$

where $[X^*] = 9.5 \times 10^{-7}[X]$, which is given by converting the unit of $[X]$ in OD to $[X^*]$ in [gDCW/μl]. The cell concentration in [gDCW/l] can be obtained by $[X^*] \times 10^6$.

1.2.18. Effects of transcription factors on main metabolic pathways

As explained in the main text, a set of metabolic pathway genes are under control of the transcription factors. In the present model, such effects of the transcription factors reflect the metabolic pathway fluxes (Fig. 1 in the main text). Specifically, the maximum velocity ($v_\bullet^{\max}$) of the enzymatic reaction may be modified as a function of the transcription factors as follows:

$$v_\bullet^{\max} = v_\bullet^{\max'} \cdot \left(1 + \sum_i TF_i\right) \cdot \prod_j \left(1 - TF_j\right) \tag{S29a}$$

where TF_i or TF_j are the activities of the transcription factor (Hardiman et al., 2010).

The maximum velocities of the Pfl and Frd reactions may be expressed as follows because those reactions are not active under aerobic cultivation, but induced as the oxygen level decreases:

$$v_{\bullet}^{\max} = v_{\bullet}^{\max'} \cdot \sum_i TF_i \cdot \prod_j \left(1 - TF_j\right) \quad \text{(S29b)}$$

The activities of the transcription factor such as cAMP-Crp, Cra, PdhR, ArcA, Fnr, and XylR may be expressed as follows:

$$TF_{cAMP-Crp} = \frac{[cAMP-Crp]^n}{[cAMP-Crp]^n + K_{cAMP-Crp}{}^n} \quad \text{(S30a)}$$

$$TF_{Cra} = \frac{[Cra]^n}{[Cra]^n + K_{Cra}{}^n} \quad \text{(S30b)}$$

$$TF_{PdhR} = \frac{[PdhR]^n}{[PdhR]^n + K_{PdhR}{}^n} \quad \text{(S30c)}$$

$$TF_{ArcA} = \frac{[Q]^n}{[Q]^n + K_{ArcA}{}^n} \quad \text{(S30d)}$$

$$TF_{Fnr} = \frac{[O_2]^n}{[O_2]^n + K_{Fnr}{}^n} \quad \text{(S30e)}$$

$$TF_{XylR} = \frac{[XYL]^n}{[XYL]^n + K_{XylR}{}^n} \quad \text{(S30f)}$$

The activity of IclR may be expressed as a function of PYR and GOX (which are antagonistic effectors of the IclR) (Lorca et al., 2007) as follows:

$$TF_{IclR} = \frac{[PYR]^n}{[PYR]^n + K_{IclR,PYR}{}^n} - \frac{[GOX]^n}{[GOX]^n + K_{IclR,GOX}{}^n} \quad \text{(S30g)}$$

Table S1 shows the effects of TFs on the primary metabolic pathways included in the present model.

Table S1. Transcription factors and its regulated genes.

TF	+/-	Metabolic pathway
cAMP-Crp	+	PTS4 (*ptsG*), NPTS (*npts*), Acs (*acs*), CS (*gltA*), αKGDH (*sucAB*), SDH (*sdhCDAB*), MDH (*mdh*), XT (*xylFGH*), Xyi (*xylA*), Xyk (*xylB*), MaeB (*maeB*), SfcA (*sfcA*), Pps (*ppsA*), Pck (*pckA*)
	-	Icl (*aceA*) [*1], MS (*aceB*) [*1]
Cra	+	ICDH (*icdA*), Icl (*aceA*), MS (*aceB*), Fbp (*fbp*), Pps (*ppsA*), Pck (*pckA*)
	-	Pfk (*pfkA*), Fba (*fba*), L_Emp (*L_emp*), Pyk (*pykF*), G6PDH (*zwf*), MaeB (*maeB*), Ppc (*ppc*)
ArcA	+	Pfl (*pflB*), Cyd (*cydAB*) , PTACK (*ptack*)
	-	PDH (*lpdA*, *aceEF*), CS (*gltA*), ICDH (*icdA*), αKGDH (*lpdA*, *sucAB*), SDH (*sdhCDAB*), Nuo (*nuoABCDEFGHIJKLMN*), Cyo (*cyoABCDE*)
Fnr	+	Pfl (*pflB*), Frd (*frdABCD*)
	-	αKGDH (*lpdA*, *sucAB*), SDH (*sdhCDAB*), Nuo (*nuoABCDEFGHIJKLMN*), Ndh (*ndh*), Cyo (*cyoABCDE*), Cyd (*cydAB*)
PdhR	-	PDH (*aceEF*, *lpdA*)
IclR	-	Icl (*aceA*), MS (*aceB*)
XylR	+	XT (*xylFGH*), Xyi (*xylA*), Xyk (*xylB*)

[*1] For the case where Cra activity is higher than cAMP-Crp activity, cAMP-Crp activity is excluded in this model because of the overriding regulatory activity of Cra over CRP (Kim et al., 2018).

1.2.19. Modeling for MVA pathways

To consider the rational design for the useful metabolite production, we incorporated the mevalonate (MVA) pathways, where MVA is formed from AcCoA through three-step reactions which correspond to the MVA1, MVA2, and MVA3. The rate reactions were expressed as (Dalwadi et al., 2018)

$$v_{MVA1} = \frac{v_{MVA1}^{\max,f}\left[AcCoA\right]}{[AcCoA] + K_{AcCoA}} - \frac{v_{MVA1}^{\max,r}\left[aAcCoA\right]}{[aAcCoA] + K_{aAcCoA}} \tag{S31a}$$

$$v_{MVA2} = \frac{v_{MVA2}^{\max}\left[AcCoA\right]\left[aAcCoA\right]}{\left[AcCoA\right]\left[aAcCoA\right] + \left(1 + \frac{\left[aAcCoA\right]}{K_i}\right)K_{AcCoA}[aAcCoA] + K_{aAcCoA}\left[AcCoA\right]} \tag{S31b}$$

$$v_{MVA3} = \frac{v_{MVA3}^{\max}\left[HMGCoA\right]}{[HMGCoA] + K_{HMGCoA}} \tag{S31c}$$

Since the NADPH is consumed by the reaction of MVA3, we modified the equation as a function of NADPH as follows:

$$v_{MVA3} = v_{MVA3}^{\max} \cdot \frac{\left[HMGCoA\right]}{[HMGCoA] + K_{HMGCoA}} \cdot \frac{\left[NADPH\right]}{[NADPH] + K_{NADPH}} \tag{S31d}$$

2. Model parameters

Table S2 and Table S3 show the kinetic parameters and the total concentrations for transcription factor and cofactor concentrations, respectively. Other parameters are also shown in Table S4.

Table S2. Model parameter values.

Reaction name	Parameter	Value	Reference
PTS1	k_1	322.3 [gDCW/gProt/s]	*1
	k_{-1}	46.3 [gDCW/gProt/s]	Kotte et al., 2010
PTS4	$v_{PTS4}^{max'}$	6.01 [μmol/gDCW/s]	*1
	$K_{EIIA\sim P}$	8.5E-3 [gProt/gDCW]	Kotte et al., 2010
	K_{Glc}	1.2E-3 [g/l]	Kotte et al., 2010
NPTS	$v_{NPTS}^{max'}$	1.1 [μmol/gDCW/s]	*1
	K_S	1.0E-2 [μmol/gDCW]	Bettenbrock et al., 2006
	K_I	5.0E-2 [μmol/gDCW]	*1
Glk	$v_{Glk}^{max'}$	18.5 [μmol/gDCW/s]	*1
	K_S	5.0 [μmol/gDCW]	*1
	K_I	8.0 [μmol/gDCW]	*1
Pgi	$v_{Pgi}^{max'}$	92.511 [μmol/gDCW/s]	*1
	K_{G6P}	4.362 [μmol/gDCW] *2	Chassagnole et al., 2002

	K_{F6P}	0.355 [μmol/gDCW] [*2]	Chassagnole et al., 2002
	$K_{G6P,6PGinh}$	0.355 [μmol/gDCW] [*2]	Chassagnole et al., 2002
	$K_{F6P,6PGinh}$	0.355 [μmol/gDCW] [*2]	Chassagnole et al., 2002
	K_{eq}	1.65	*1
Pfk	$v_{Pfk}^{max'}$	92.511 [μmol/gDCW/s]	*1
	K_{F6P}	2.2E-2 [μmol/gDCW]	Kotte et al., 2010
	K_{PEP}	0.138 [μmol/gDCW]	Kotte et al., 2010
	L	9.5E+7	Kotte et al., 2010
	n	4	Kotte et al., 2010
Fba	$v_{Fba}^{max',f}$	10.195 [μmol/gDCW/s]	*1
	$v_{Fba}^{max',r}$	10.195 [μmol/gDCW.s]	*1
	K_{FBP}	5.92 [μmol/gDCW]	Kotte et al., 2010
	$K_{GAP/DHAP}$	16.6 [μmol/gDCW]	Kotte et al., 2010
L_Emp	$v_{L\_Emp}^{max',f}$	10.195 [μmol/gDCW/s]	*1
	$v_{L\_Emp}^{max',r}$	10.195 [μmol/gDCW.s]	*1
	$K_{GAP/DHAP}$	4.76 [μmol/gDCW]	Kotte et al., 2010
	K_{PEP}	1.11 [μmol/gDCW]	Kotte et al., 2010
Pyk	$v_{Pyk}^{max'}$	49.502 [μmol/gDCW/s]	*1
	K_{PEP}	5.0 [μmol/gDCW]	Kotte et al., 2010

	K_{FBP}	0.413 [μmol/gDCW]	Kotte et al., 2010
	L	1.0E+5	Kotte et al., 2010
	n	4	Kotte et al., 2010
G6PDH	$v_{G6PDH}^{\max*}$	14.3 [μmol/gDCW/s]	*1
	k_{G6PDH}	9.886E+3 [s]	Yao et al., 2011
	$k_{G6PDH}^{'}$	0.356 [-]	Yao et al., 2011
	K_{G6P}	10.2 [μmol/gDCW]	*1
	K_{NADP}	0.044 [μmol/gDCW] *2	Chassagnole et al., 2002
	K_{NADPH_G6Pinh}	11.401 [μmol/gDCW] *2	Chassagnole et al., 2002
	$K_{NADPH\_NADPinh}$	0.018 [μmol/gDCW] *2	Chassagnole et al., 2002
PGDH	$v_{PGDH}^{\max*}$	28.781 [μmol/gDCW/s]	*1
	k_{PGDH}	4.271E+3 [s]	Yao et al., 2011
	$k_{PGDH}^{'}$	0.886 [-]	Yao et al., 2011
	K_{6PG}	11.3 [μmol/gDCW]	*1
	K_{NADP}	0.090 [μmol/gDCW] *2	Chassagnole et al., 2002
	$K_{NADPHinh}$	0.025 [μmol/gDCW] *2	Chassagnole et al., 2002
	K_{ATPinh}	368.794 [μmol/gDCW] *2	Chassagnole et al., 2002
Rpe	$v_{Rpe}^{\max'}$	11.949 [μmol/gDCW/s] *2	Chassagnole et al., 2002
	K_{eq}	1.4	Chassagnole et al., 2002

Rpi	$v_{Rpi}^{max'}$	8.579 [μmol/gDCW/s] *2	Chassagnole et al., 2002
	K_{eq}	4.0	Chassagnole et al., 2002
TktA	$v_{TktA}^{max'}$	16.797 [μmol/gDCW/s] *2	Chassagnole et al., 2002
	K_{eq}	1.2	Chassagnole et al., 2002
TktB	$v_{TktB}^{max'}$	76.736 [μmol/gDCW/s] *2	Chassagnole et al., 2002
	K_{eq}	10.0	Chassagnole et al., 2002
Tal	$v_{Tal}^{max'}$	19.276 [μmol/gDCW/s] *2	Chassagnole et al., 2002
	K_{eq}	1.05	Chassagnole et al., 2002
XT	$v_{XT}^{max'}$	26.7 [μmol/gDCW/s]	*1
	K_{XYL}	1.4 [g/l]	*1
	K_{I}	0.01 [gProt/gDCW]	*1
Xyi	$v_{Xyi}^{max'}$	14.628 [μmol/gDCW/s]	*1
	$K_{XYL^{in}}$	17.731 [μmol/gDCW] *2	Altintas et al., 2006
Xyk	$v_{Xyk}^{max'}$	12.283 [μmol/gDCW/s]	*1
	K_{XYLU}	1.418 [μmol/gDCW] *2	Altintas et al., 2006
	K_{ATP}	0.709 [μmol/gDCW] *2	Altintas et al., 2006
Edd	$v_{Edd}^{max'}$	0.3 [μmol/gDCW/s]	*1
	K_{6PG}	1.064 [μmol/gDCW] *2	Peskov et al., 2012
	K_{KDPG}	1.773 [μmol/gDCW] *2	Peskov et al., 2012

	K_{eq}	1.0E+3 [-]	Peskov et al., 2012
	pH_m	6.4	Peskov et al., 2012
	pK	10	Peskov et al., 2012
Eda	$v_{Edd}^{max'}$	2.0 [μmol/gDCW/s]	*1
	K_{KDPG}	0.621 [μmol/gDCW] *2	Peskov et al., 2012
	K_{PYR}	17.731 [μmol/gDCW] *2	Peskov et al., 2012
	$K_{GAP/DHAP}$	1.773 [μmol/gDCW] *2	Peskov et al., 2012
	K_{eq}	28.0 [-]	*1
	pH_m	7.5	Peskov et al., 2012
	pK	10.0	Peskov et al., 2012
LDH	$v_{LDH}^{max'}$	51.7 [μmol/gDCW/s]	*1
	K_{NADH}	0.9 [μmol/gDCW]	*1
	K_{PYR}	26.7 [μmol/gDCW]	*1
	n_{NADH}	3	*1
	n_{PYR}	2	*1
Pfl	$v_{Pfl}^{max',f}$	14.7 [μmol/gDCW/s]	*1
	$v_{Pfl}^{max',r}$	3.4 [μmol/gDCW.s]	*1
	K_{PYR}	4.5 [μmol/gDCW]	*1
	K_{CoA}	0.782 [μmol/gDCW]	*1
	K_{AcCoA}	5.862 [μmol/gDCW]	*1
	K_{FOR}	1.128 [g/l]	Cintolesi et al., 2012

ALDH	$v_{ALDH}^{max'}$	63.9 [μmol/gDCW/s]	*1
	K_{AcCoA}	0.012 [μmol/gDCW] *2	Hoefnagel et al., 2002
	K_{NADH}	0.443 [μmol/gDCW]	*1
	K_{NAD}	0.142 [μmol/gDCW] *2	Hoefnagel et al., 2002
	K_{CoA}	0.014 [μmol/gDCW] *2	Hoefnagel et al., 2002
	K_{ACAL}	17.731 [μmol/gDCW] *2	Hoefnagel et al., 2002
	K_{eq}	27.73 [μmol/gDCW]	*1
ADH	$v_{ADH}^{max'}$	57.97 [μmol/gDCW/s]	*1
	K_{ACAL}	0.053 [μmol/gDCW] *2	Hoefnagel et al., 2002
	K_{NADH}	2.1 [μmol/gDCW]	*1
	K_{NAD}	0.142 [μmol/gDCW] *2	Hoefnagel et al., 2002
	K_{ETH}	7.107E-04 [g/l]	*1
	K_{eq}	2.191E+04 [μmol/gDCW] *2	Hoefnagel et al., 2002
PTACK	$v_{PTACK}^{max'}$	1.8 [μmol/gDCW/s]	*1
	K_{AcCoA}	2.2E-2 [μmol/gDCW]	Kotte et al., 2010
	K_{PYR}	2.2E-2 [μmol/gDCW]	Kotte et al., 2010
	L	6.39E+5	Kotte et al., 2010
	n	2	Kotte et al., 2010
Acs	$v_{Acs}^{max'}$	0.729 [μmol/gDCW/s]	*1

	K_{ACT}	0.1 [g/l]	*1
PDH	$v_{PDH}^{\max'}$	5.667 [μmol/gDCW/s]	*1
	K_{PYR}	0.128 [μmol/gDCW]	Kotte et al., 2010
	K_{GOX}	0.218 [μmol/gDCW]	Kotte et al., 2010
	$K_{I,PYR}$	0.231 [μmol/gDCW]	Kotte et al., 2010
	L	3.4	Kotte et al., 2010
	n	2.65	Kotte et al., 2010
CS	$v_{CS}^{\max'}$	2.115 [μmol/gDCW/s]	*1
	K_{AcCoA}	0.212 [μmol/gDCW]	Kotte et al., 2010
	K_{OAA}	0.029 [μmol/gDCW]	Kotte et al., 2010
	$K_{OAA,AcCoA}$	0.029 [μmol/gDCW]	Kotte et al., 2010
	$K_{\alpha KG}$	0.63 [μmol/gDCW]	Kotte et al., 2010
ICDH	$v_{ICDH}^{\max'}$	7.887 [μmol/gDCW/s]	*1
	$K_{CIT/ICIT}$	1.6E-4 [μmol/gDCW]	Kotte et al., 2010
	K_{PEP}	0.334 [μmol/gDCW]	Kotte et al., 2010
	L	127.0	Kotte et al., 2010
	n	2	Kotte et al., 2010
KGDH	$v_{KGDH}^{\max'}$	1.183 [μmol/gDCW/s]	*1
	$K_{\alpha KG}$	0.548 [μmol/gDCW]	Kotte et al., 2010
SDH/Frd	$v_{SDH}^{\max'}$	1.810 [μmol/gDCW/s]	*1

	$v_{Frd}^{\max'}$	2.5 [μmol/gDCW/s]	*1
	K_{SUC}	0.230 [μmol/gDCW]	*1
	$K_{FUM/MAL}$	0.575 [μmol/gDCW]	*1
MDH	$v_{MDH}^{\max',f}$	12.677 [μmol/gDCW/s]	*1
	$v_{MDH}^{\max',r}$	54.332 [μmol/gDCW/s]	*1
	$K_{FUM/MAL}$	4.610 [μmol/gDCW] *2	Usuda et al., 2010
	K_{NAD}	0.461 [μmol/gDCW] *2	Usuda et al., 2010
	K_{OAA}	0.087 [μmol/gDCW] *2	Usuda et al., 2010
	K_{NADH}	0.108 [μmol/gDCW] *2	Usuda et al., 2010
Ppc	$v_{Ppc}^{\max'}$	1.7 [μmol/gDCW/s]	*1
	K_{PEP}	0.048 [μmol/gDCW]	Kotte et al., 2010
	K_{FBP}	0.408 [μmol/gDCW]	Kotte et al., 2010
	K_{AcCoA}	0.5 [μmol/gDCW]	*1
	L	5.2E+6	Kotte et al., 2010
	n	3	Kotte et al., 2010
Icl	$v_{Icl}^{\max'}$	6.7 [μmol/gDCW/s]	*1
	$K_{CIT/ICIT}$	0.0022 [μmol/gDCW]	*1
	K_{PEP}	1.55 [μmol/gDCW]	*1
	$K_{GAP/\sim/2PG}$	1.44 [μmol/gDCW]	*1

	$K_{\alpha KG}$	0.827 [μmol/gDCW]	Kotte et al., 2010
	L	5.01	*[1]
	n	2	*[1]
MS	$v_{MS}^{max'}$	7.8 [μmol/gDCW/s]	*[1]
	K_{GOX}	0.95 [μmol/gDCW]	Kotte et al., 2010
	K_{AcCoA}	0.755 [μmol/gDCW]	Kotte et al., 2010
	$K_{GOXAcCoA}$	0.719 [μmol/gDCW]	Kotte et al., 2010
Fbp	$v_{Fbp}^{max'}$	0.114 [μmol/gDCW/s]	*[1]
	K_{FBP}	3.0E-3 [μmol/gDCW]	Kotte et al., 2010
	K_{PEP}	0.3 [μmol/gDCW]	Kotte et al., 2010
	L	4.0E+6	Kotte et al., 2010
	n	4	Kotte et al., 2010
Pps	$v_{Pps}^{max'}$	4.1E-3 [μmol/gDCW/s]	*[1]
	K_{PYR}	1.77E-3 [μmol/gDCW]	Kotte et al., 2010
	K_{PEP}	1.0E-3 [μmol/gDCW]	Kotte et al., 2010
	L	1.0E-79	Kotte et al., 2010
	n	2	Kotte et al., 2010
Pck	$v_{Pck}^{max'}$	0.087 [μmol/gDCW/s]	*[1]
	K_{OAA}	0.184 [μmol/gDCW]	Kotte et al., 2010
	K_{PEP}	1.0E+3 [μmol/gDCW]	Kotte et al., 2010
MaeB	$v_{MaeB}^{max'}$	0.44 [μmol/gDCW/s]	*[1]

	K_{MAL}	9.929 [μmol/gDCW] *2	Usuda et al., 2010
	$K_{NADP^{+}}$	0.027 [μmol/gDCW] *2	Usuda et al., 2010
	n	1.0	Usuda et al., 2010
SfcA	$v_{SfcA}^{max'}$	0.03 [μmol/gDCW/s]	*1
	K_{MAL}	0.337 [μmol/gDCW] *2	Usuda et al., 2010
	$K_{NAD^{+}}$	0.082 [μmol/gDCW] *2	Usuda et al., 2010
	n	1.0	Usuda et al., 2010
SUC_{trans}	$v_{SUC_{trans}}^{max'}$	0.5 [μmol/gDCW/s]	*1
	K_{SUC}	16.0 [μmol/gDCW]	*1
Cya	$v_{Cya}^{max'}$	0.993 [μmol/gDCW/s]	*1
	$K_{EIIA\sim P}$	4.7E-3 [gProt/gDCW]	*1
cAMPdegr	$v_{Degr}^{max'}$	1.0 [μmol/gDCW/s]	*1
	K_{cAMP}	0.1 [μmol/gDCW]	Kotte et al., 2010
cAMP-Crp association/ dissociation	$p_{cAMP-Crp}$	1.0E+8 [/s]	Kotte et al., 2010
	K_{cAMP}	0.895 [μmol/gDCW]	Kotte et al., 2010
	n	1	Kotte et al., 2010
Cra-FBP association/ dissociation	$p_{Cra-FBP}$	100 [/s]	Kotte et al., 2010
	K_{FBP}	1.36 [μmol/gDCW]	Kotte et al., 2010
	n	2	Kotte et al., 2010
PdhR-PYR association/	$p_{PdhR-PYR}$	100 [/s]	Kotte et al., 2010

dissociation			
	K_{PYR}	0.164 [μmol/gDCW]	Kotte et al., 2010
	n	1	Kotte et al., 2010
Nuo	$v_{Nuo}^{\max'}$	708.333 [μmol/gDCW/s]	*1
	$K_{m,Nuo,Q}$	0.34 [μmol/gDCW]	Henkel et al., 2014
	$K_{m,Nuo,NADH}$	20 [μmol/gDCW]	Henkel et al., 2014
Ndh	$v_{Ndh}^{\max'}$	11.111 [μmol/gDCW/s]	*1
	$K_{m,Ndh,Q}$	0.34 [μmol/gDCW]	Henkel et al., 2014
	$K_{m,Ndh,NADH}$	20 [μmol/gDCW]	Henkel et al., 2014
Cyo	$v_{Cyo}^{\max'}$	800.0 [μmol/gDCW/s]	*1
	K_{m,Cyo,O_2}	2E-1 [mM]	*1
	K_{m,Cyo,QH_2}	25.0 [μmol/gDCW]	*1
Cyd	$v_{Cyd}^{\max'}$	31.558 [μmol/gDCW/s]	*1
	K_{m,Cyd,O_2}	2.4E-2 [mM]	*1
	K_{m,Cyd,QH_2}	10.0 [μmol/gDCW]	*1
QH_2 synthesis	p_{syn,QH_2}	2.16 [μmol/gDCW]	*1
G6P synthesis	$k_{Bio,G6P}^{+}$	554.4 [-]	*1
	$k_{Bio,G6P}^{++}$	0.002 [/s]	*1
F6P synthesis	$k_{Bio,F6P}^{+}$	554.4 [-]	*1

	$k^{++}_{Bio,F6P}$	0.002 [/s]	*1
GAP/DHAP synthesis	$k^{+}_{Bio,GAP/DHAP}$	352.8 [-]	*1
	$k^{++}_{Bio,GAP/DHAP}$	0.002 [/s]	*1
PEP synthesis	$k^{+}_{Bio,PEP}$	3.046E+3	*1
	$k^{++}_{Bio,PEP}$	0.002 [/s]	*1
PYR synthesis	$k^{+}_{Bio,PYR}$	39.816 [-]	*1
	$k^{++}_{Bio,PYR}$	0.002 [/s]	*1
AcCoA synthesis	$k^{+}_{Bio,AcCoA}$	135.36 [-]	*1
	$k^{++}_{Bio,AcCoA}$	0.035 [/s]	*1
R5P synthesis	$k^{+}_{Bio,R5P}$	72.0 [-]	*1
	$k^{++}_{Bio,R5P}$	0.002 [/s]	*1
E4P synthesis	$k^{+}_{Bio,E4P}$	72.0 [-]	*1
	$k^{++}_{Bio,E4P}$	0.002 [/s]	*1
αKG synthesis	$k^{+}_{Bio,\alpha KG}$	7.042E+3 [-]	*1
	$k^{++}_{Bio,\alpha KG}$	0.002 [/s]	*1
OAA synthesis	$k^{+}_{Bio,OAA}$	4.608E+4 [-]	*1
	$k^{++}_{Bio,OAA}$	0.002 [/s]	*1
cAMP-Crp	$K_{cAMP-Crp}$	5.0E-3 [gProt/gDCW]	*1

	n	4	*1
Cra	K_{Cra}	1.5E-3 [gProt/gDCW]	*1
	n	1	*1
PdhR	K_{PdhR}	1.0E-3 [gProt/gDCW]	*1
	n	1	*1
IclR	$K_{IclR,PYR}$	1.4 [μmol/gDCW]	*1
	$K_{IclR,GOX}$	7.9 [μmol/gDCW]	*1
	n	3	*1
ArcA	K_{ArcA}	0.5 [μmol/gDCW]	*1
	n	-5	*1
Fnr	K_{Fnr}	1.0E-2 [mM]	*1
	n	-5	*1
MVA1	$v_{MVA1}^{\max,f}$	6.5 [μmol/gDCW/s]	*1
	$v_{MVA1}^{\max,r}$	10.0 [μmol/gDCW/s]	*1
	K_{AcCoA}	0.564 [μmol/gDCW] *2	Dalwadi et al., 2018
	K_{aAcCoA}	5.64E-3 [μmol/gDCW] *2	Dalwadi et al., 2018
MVA2	$v_{MVA2}^{\max}$	1.55 [μmol/gDCW/s]	*1
	K_{AcCoA}	8.5E-3 [μmol/gDCW] *2	Dalwadi et al., 2018
	K_{aAcCoA}	1.7E-3 [μmol/gDCW] *2	Dalwadi et al., 2018
	K_i	5.64E-3 [μmol/gDCW] *2	Dalwadi et al., 2018
MVA3	$v_{MVA3}^{\max}$	4.6 [μmol/gDCW/s]	*1
	K_{HMGCoA}	1.13E-2 [μmol/gDCW] *2	Dalwadi et al., 2018
	K_{NADPH}	0.5 [μmol/gDCW]	*1

*1 The parameter values were tuned in the present model.

*2 The values were converted into [μmol/gDCW] using cell density 564 [gDCW/L cell

volume] (Chassagnole et al. 2002).

Table S3. Total concentrations for transcription factor and cofactor concentrations.

Concentration	Value	Reference
$[Crp]_{total}$	7.29E-3 [gProt/gDCW]	Kotte et al., 2010
$[Cra]_{total}$	7.29E-3 [gProt/gDCW]	Kotte et al., 2010
$[PdhR]_{total}$	7.29E-3 [gProt/gDCW]	Kotte et al., 2010
$[IclR]$	7.29E-3 [gProt/gDCW]	Kotte et al., 2010
$[ATP]$	7.571 [μmol/gDCW] [*1]	Chassagnole et al., 2002
$[NADP]$	0.13 [μmol/gDCW]	Pramanik and Keasling, 1997
$[NA*]_{total}$	12.90 [μmol/gDCW]	Berrios-Rivera et al., 2002
$[CoA]$	0.887 [μmol/gDCW]	assumed

[*1] The values were converted into [μmol/gDCW] using cell density 564 [gDCW/L cell volume] (Chassagnole et al. 2002).

Table S4. Other parameter values.

	Value	Reference
k_{ATP}	9.4E-6 [gDCW/μmol]	*[1]
k_{NADPH}	9.081 [/s]	*[1]
k_{ACT}	0.6 [g/l]	*[1]
$[O_2]_{Saturation}$	0.214 [mM]	-
pH	7.5	Peskov et al., 2012
M_w^{GLC}	180.156	-
M_w^{XYL}	150.130	-
M_w^{ACT}	60.050	-
M_w^{LAC}	90.08	-
M_w^{FOR}	46.025	-
M_w^{ETH}	46.07	-
M_w^{SUC}	118.09	
p_{uc}	9.5E-7 [gDCW/μ(OD).l]	Kotte et al., 2010

*[1] The parameter values were tuned in the present model.

3. Supplementary results

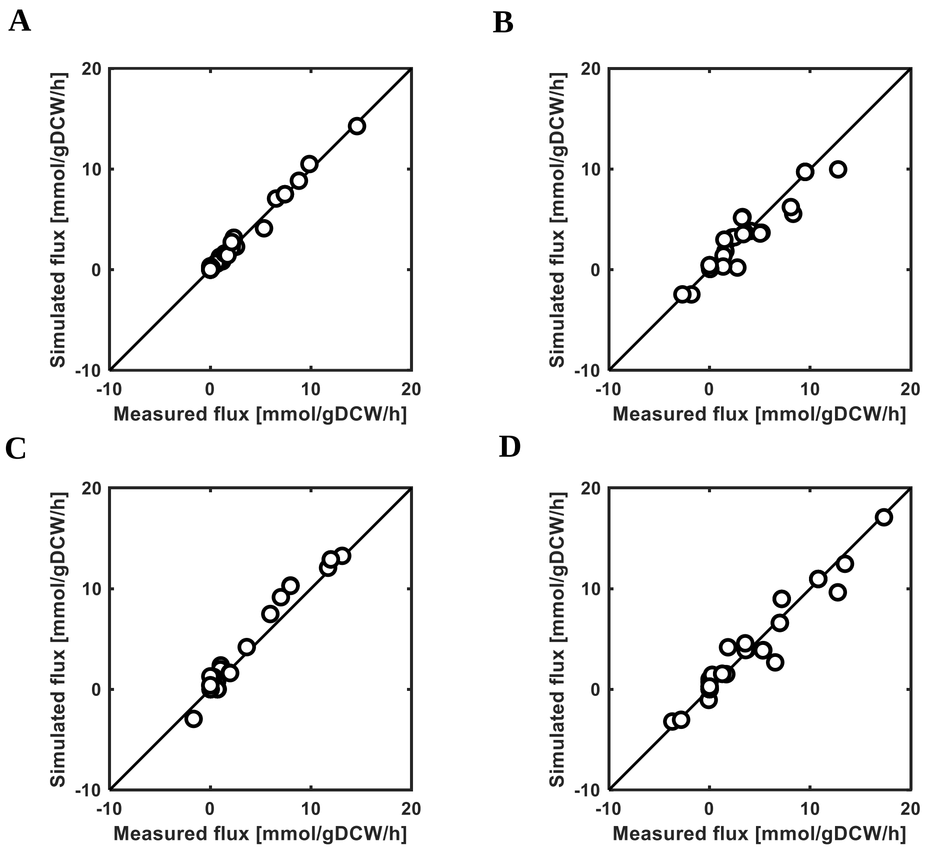

Figure S1. Comparison of the simulated values with the experimental data in wild-type *E. coli*. For the cases of using glucose under aerobic condition (A), xylose under aerobic condition (B), glucose under anaerobic condition (C), and xylose under anaerobic condition (D). The experimental data were taken from Gonzalez et al. (2017).

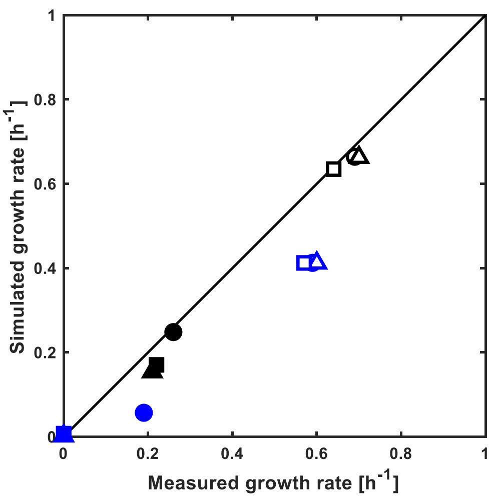

Figure S2. Comparison of the simulated values with the experimental data in wild-type strain, and *pfl*- and *ack*- knockout mutants. Open and filled symbols indicate aerobic and anaerobic conditions, respectively. Black and blue colors indicate the case of using glucose and xylose as a carbon source, respectively. Circle, triangle, and square symbols indicate the wild-type strain, and *pfl*- and *ack*- knockout mutants, respectively. The experimental data of the specific growth rate were taken from Hasona et al. (2004).

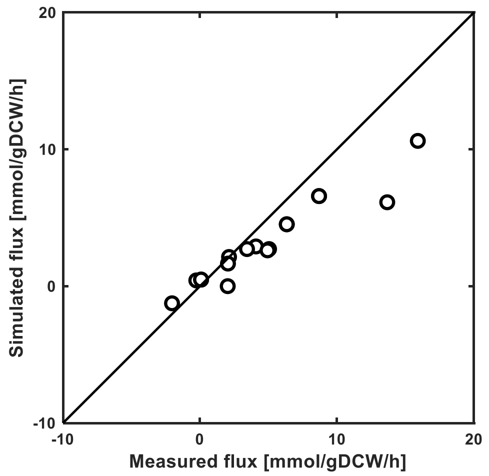

Figure S3. Comparison of the simulated fluxes with the experimental ones in *ptsG*-knockout mutant grown on glucose and xylose under aerobic condition. The experimental data were taken from Long et al. (2016).

Table S5. Comparison of the simulated values with the experimental data in wild-type *E. coli* and single-gene knockout mutants for the continuous culture. The experimental data were taken from Ishii et al. (2007).

	Dilution rate [h^{-1}]	Correlation coefficient	p-value
Δ*glk*	0.2	0.92	1.4E-11
Δ*pgi*	0.2	0.87	1.3E-09
Δ*pfkA*	0.2	0.89	3.8E-10
Δ*pfkB*	0.2	0.93	4.2E-12
Δ*fbp*	0.2	0.90	9.3E-11
Δ*fbaB*	0.2	0.92	7.0E-12
Δ*gapC*	0.2	0.93	1.7E-12
Δ*gpmA*	0.2	0.92	2.1E-11
Δ*gpmB*	0,2	0.93	1.6E-12
Δ*pykA*	0.2	0.93	3.9E-12
Δ*pykF*	0.2	0.91	3.5E-11
Δ*ppsA*	0.2	0.92	5.7E-12
Δ*zwf*	0.2	0.96	2.1E-15
Δ*gnd*	0.2	-	-
Δ*rpe*	0.2	0.93	9.2E-13
Δ*rpiA*	0.2	0.96	7.6E-17
Δ*rpiB*	0.2	0.95	2.7E-14
Δ*tktA*	0.2	0.93	4.9E-13
Δ*tktB*	0.2	0.91	7.2E-11
Δ*talA*	0.2	0.90	1.0E-10
Δ*talB*	0.2	0.91	5.4E-11
WT	0.1	0.90	1.7E-10
WT	0.2	0.93	2.5E-12
WT	0.4	0.97	2.1E-15
WT	0.5	0.92	1.2E-10
WT	0.7	-	-

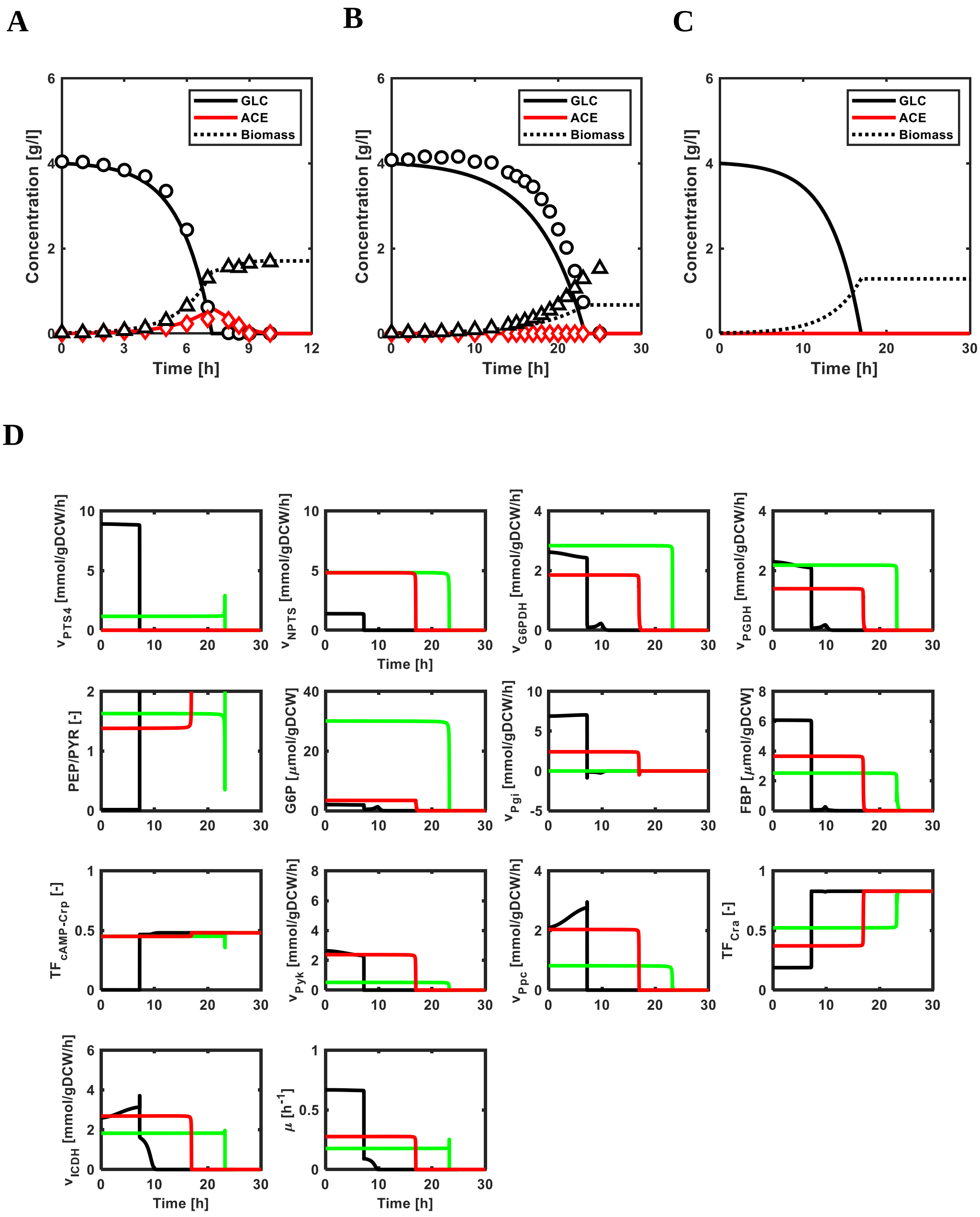

Figure S4. Simulation results of the batch cultivations of the wild type, and *pgi*- and *ptsG*- knockout mutants grown on glucose under aerobic condition. The lines and symbols represent the simulated and experimental data (Toya et al., 2010) for the time courses of extracellular metabolites and biomass concentrations of wild type (A), and *pgi*- (B) and *ptsG*- (C) knockout mutants. The black, green, and red lines represent the simulated time courses of the intracellular metabolic changes for the cases of the wild type, *pgi* mutant, and *ptsG* mutant, respectively (D).

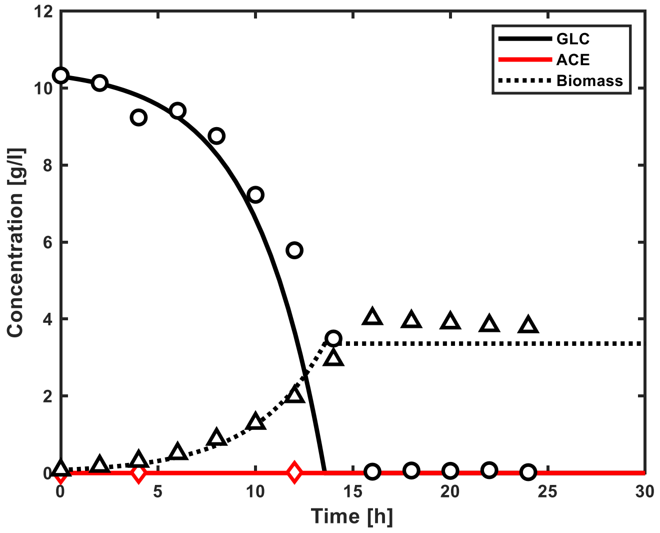

Figure S5. Simulation result of the batch cultivation using the *ptsG*-knockout mutant under aerobic condition. Time courses of extracellular metabolites and biomass concentrations, where the lines show simulation results and the symbols represent experimental data: open circle glucose; open diamond acetate; open triangle biomass. The experimental data were taken from Matsuo (2011).

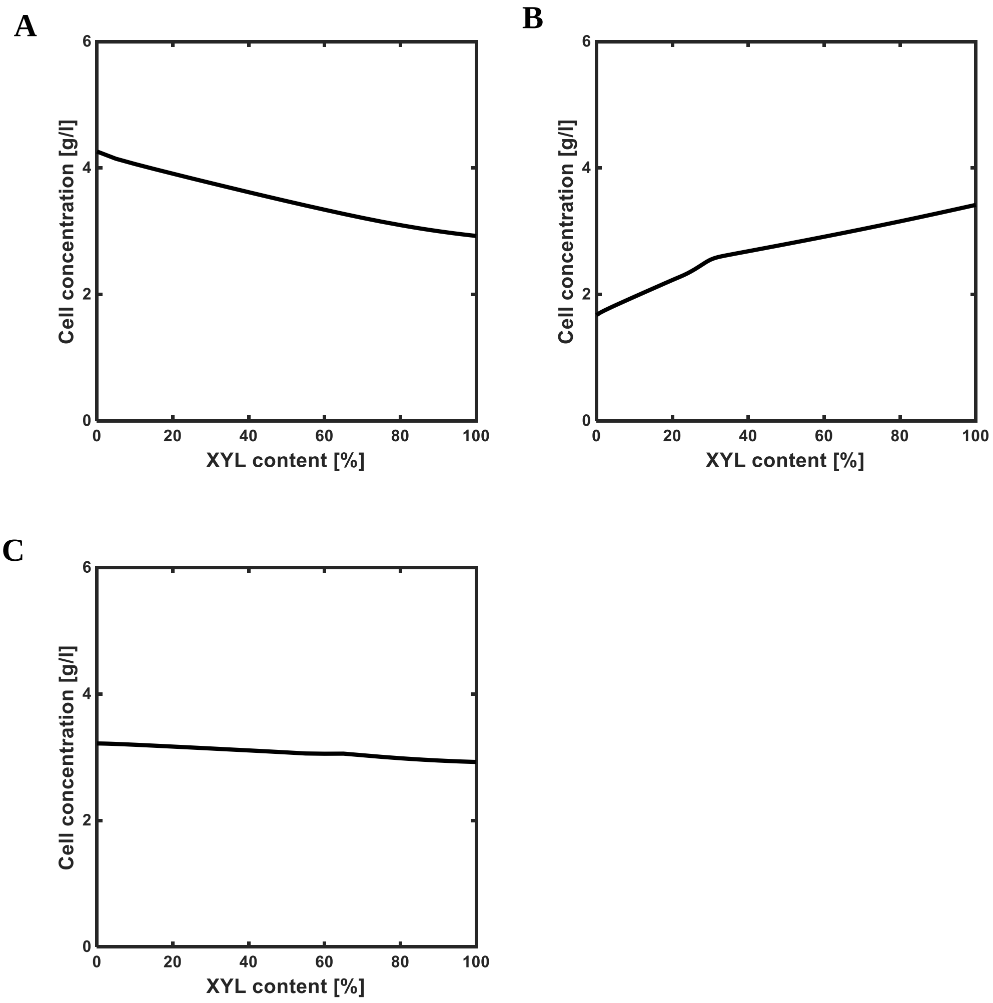

Figure S6. Effect of a ratio of the glucose and xylose on the cell concentration in the wild-type strain (A), and *pgi*-knockout (B) and *ptsG*-knockout (C) mutants. The xylose content [%] is defined as the ratio of xylose contained in a mixture. The total substrate concentration is 10 g/l.

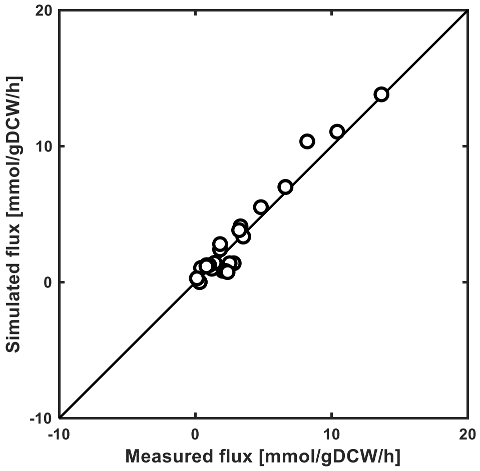

Figure S7. Comparison of the simulated fluxes with the experimental data in MVA-producing strain of *E. coli* grown on glucose under aerobic condition. The experimental data were taken from Wada et al. (2017).

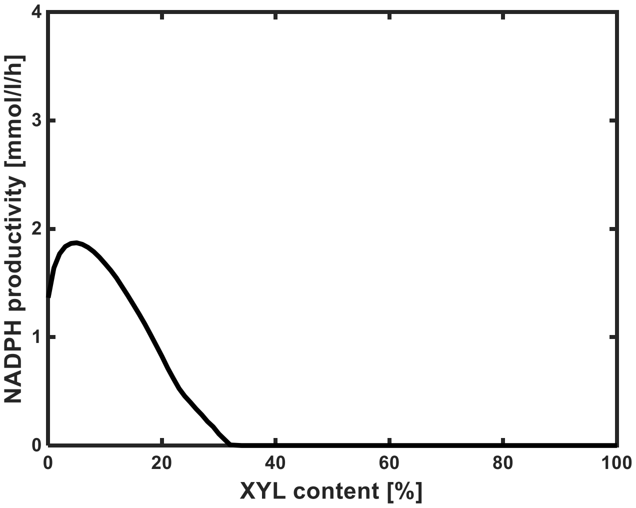

Figure S8. Effect of the change in xylose content on the NADPH productivity in the *pgi* mutant with MVA-producing pathway grown on a mixture of glucose and xylose. The xylose content [%] is defined as the ratio of xylose contained in a mixture of glucose and xylose. The total substrate concentration is 10 g/l.

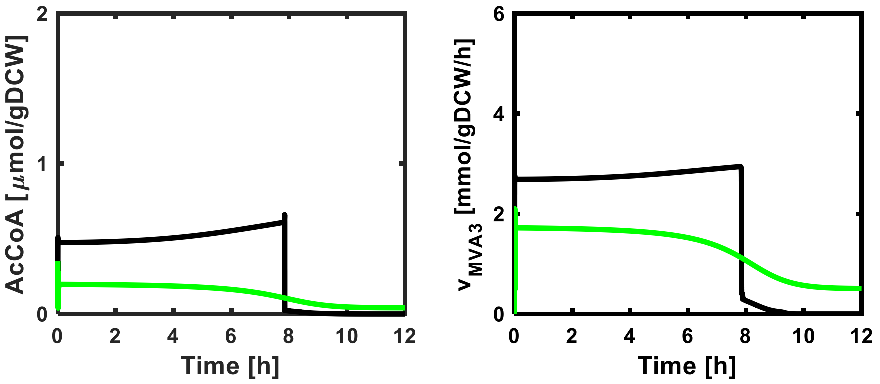

Figure S9. Simulated time courses of the intracellular metabolic changes in the MVA-producing strain. The black and green lines represent the cases of the MVA-producing *E. coli* grown on glucose, and its *pgi* mutant grown on a mixture of glucose and xylose, respectively. For the *pgi* mutant, a xylose content of 5% is used that gives the maximum productivity of NADPH, as shown in **Fig. S6**. The total substrate concentration is 4 g/l.

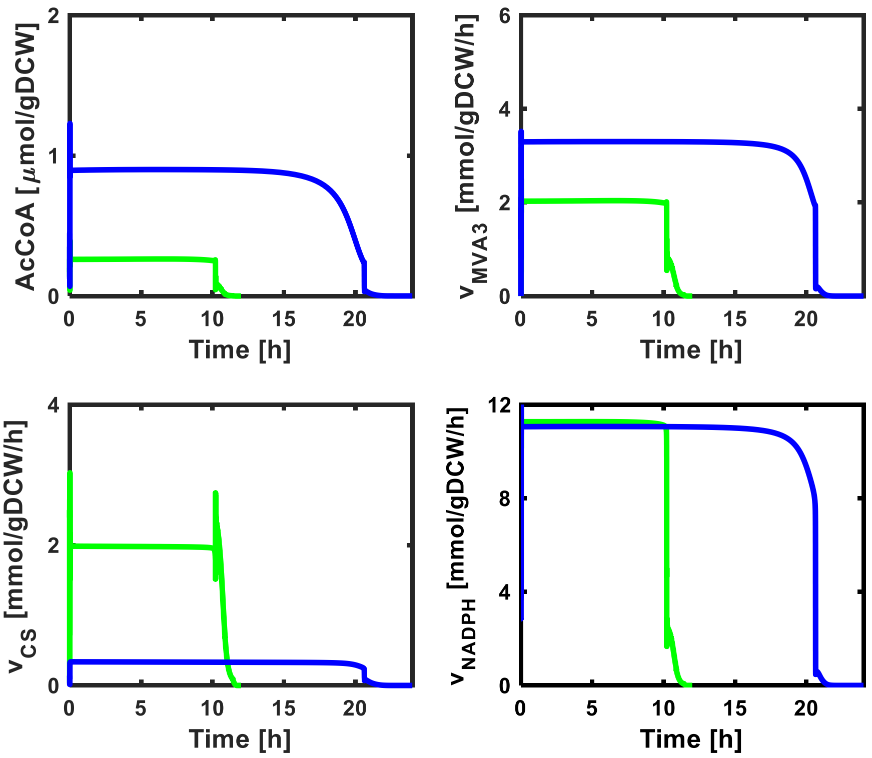

Figure S10. Simulated time courses of the intracellular metabolic changes of the MVA-producing strain for the case of using a mixture of glucose and xylose. The green and blue lines represent the cases without and with overexpression of ArcA (TF_{ArcA} = 0.95) in the *pgi* mutant with the MVA pathway, respectively. A xylose content of 33% is used, which gives the maximum MVA concentration, as shown in **Fig. 5B**. The total substrate concentration is 4 g/l.

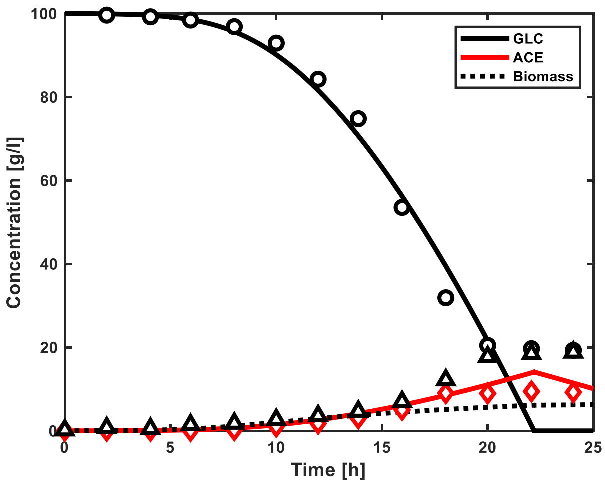

Figure S11. Simulation result of the batch cultivation with a high initial glucose concentration under aerobic condition. Time courses of extracellular metabolites and biomass concentrations, where the lines show simulation results and the symbols represent experimental data: open circle glucose; open diamond acetate; open triangle biomass. The experimental data were taken from Borja et al. (2012).

References

Alexeeva, S., Hellingwerf, K. J., Teixeira de Mattos, M. J., Quantitative assessment of oxygen availability: Perceived aerobiosis and its effect on flux distribution in the respiratory chain of *Escherichia coli*. J. Bacteriol. 2002, 184, 1402-1406.

Altintas, M. M., Eddy, C. K., Zhang, M., McMillan, J. D., Kompala, D. S., Kinetic modeling to optimize pentose fermentation in *Zymomonas mobilis*. Biotechnol. Bioeng. 2006, 94, 273-295.

Berrios-Rivera, S. J., Bennett, G. N., San, K. Y., The effect of increasing NADH availability on the redistribution of metabolic fluxes in *Escherichia coli* chemostat cultures. Metab. Eng. 2002, 4, 230-237.

Bettenbrock, K., Fischer, S., Kremling, A., Jahreis, K., Sauter, T., Gilles, E. D., A quantitative approach to catabolite repression in *Escherichia coli*. J. Biol. Chem. 2006, 281, 2578-2584.

Borja, G. M., Meza Mora, E., Barron, B., Gosset, G., Ramirez, O. T., Lara, A. R., Engineering *Escherichia coli* to increase plasmid DNA production in high cell-density cultivations in batch mode. Microb. Cell Fact. 2012, 11, 132.

Chassagnole, C., Noisommitt-Rizzi, N., Schmid, J. W., Mauch, K., Reuss, M., Dynamic modeling of the central carbon metabolism of *Escherichia coli*. Biotechnol. Bioeng. 2002, 79, 53–73.

Christodoulou, D., Link, H., Fuhrer, T., Kochanowski, K., Gerosa, L., Sauer, U., Reserve flux capacity in the pentose phosphate pathway enables *Escherichia coli*'s rapid response to oxidative stress. Cell Syst. 2018, 6, 569-578.

Cintolesi, A., Clomburg, J. M., Rigou, V., Zygourakis, K., Gonzalez, R., Quantitative analysis of the fermentative metabolism of glycerol in *Escherichia coli*. Biotechnol. Bioeng. 2012, 109, 187-198.

Dalwadi, M. P., Garavaglia, M., Webb, J. P., King, J. R., Minton, N. P., Applying asymptotic methods to synthetic biology: Modelling the reaction kinetics of the

mevalonate pathway. J. Theor. Biol. 2018, 439, 39-49.

Dearriaga, D., Soler, J., Cadenas, E., Influence of ph on the allosteric properties of lactate-dehydrogenase activity of *Phycomyces blakesleeanus*. Biochem. J. 1982, 203, 393-400.

Gonzalez, J. E., Long, C. P., Antoniewicz, M. R., Comprehensive analysis of glucose and xylose metabolism in *Escherichia coli* under aerobic and anaerobic conditions by ^{13}C metabolic flux analysis. Metab. Eng. 2017, 39, 9-18.

Hardiman, T., Meinhold, H., Hofmann, J., Ewald, J. C., Siemann-Herzberg, M., Reuss, M., Prediction of kinetic parameters from DNA-binding site sequences for modeling global transcription dynamics in *Escherichia coli*. Metab. Eng. 2010, 12, 196-211.

Hasona, A., Kim, Y., Healy, F. G., Ingram, L. O., Shanmugam, K. T., Pyruvate formate lyase and acetate kinase are essential for anaerobic growth of *Escherichia coli* on xylose. J. Bacteriol. 2004, 186, 7593-7600.

Henkel, S. G., Ter Beek, A., Steinsiek, S., Stagge, S., Bettenbrock, K., de Mattos, M. J. T., Sauter, T., Sawodny, O., Ederer, M., Basic regulatory principles of *Escherichia coli*'s electron transport chain for varying oxygen conditions. PLoS ONE 2014, 9, e107640.

Hoefnagel, M. H. N., Starrenburg, M. J. C., Martens, D. E., Hugenholtz, J., Kleerebezem, M., Van Swam, I. I., Bongers, R., Westerhoff, H. V., Snoep, J. L., Metabolic engineering of lactic acid bacteria, the combined approach: kinetic modelling, metabolic control and experimental analysis. Microbiology 2002, 148, 1003-1013.

Ishii, N., Nakahigashi, K., Baba, T., Robert, M., Soga, T., Kanai, A., Hirasawa, T., Naba, M., Hirai, K., Hoque, A., Ho, P. Y., Kakazu, Y., Sugawara, K., Igarashi, S., Harada, S., Masuda, T., Sugiyama, N., Togashi, T., Hasegawa, M., Takai, Y., Yugi, K., Arakawa, K., Iwata, N., Toya, Y., Nakayama, Y., Nishioka, T., Shimizu, K., Mori, H., Tomita, M., Multiple high-throughput analyses monitor the response of *E. coli* to perturbations. Science 2007, 316, 593-597.

Kim, D., Seo, S. W., Gao, Y., Nam, H., Guzman, G. I., Cho, B. K., Palsson, B. O., Systems assessment of transcriptional regulation on central carbon metabolism by Cra

and CRP. Nucleic Acids Res. 2018, 46, 2901-2917.

Kotte, O., Zaugg, J. B., Heinemann, M., Bacterial adaptation through distributed sensing of metabolic fluxes. Mol. Syst. Biol. 2010, 6, 355.

Kremling, A., Bettenbrock, K., Laube, B., Jahreis, K., Lengeler, J. W., Gilles, E. D., The organization of metabolic reaction networks. III. Application for diauxic growth on glucose and lactose. Metab. Eng. 2001, 3, 362-379.

Long, C. P., Au, J., Gonzalez, J. E., Antoniewicz, M. R., ^{13}C metabolic flux analysis of microbial and mammalian systems is enhanced with GC-MS measurements of glycogen and RNA labeling. Metab. Eng. 2016, 38, 65-72.

Lorca, G. L., Ezersky, A., Lunin, V. V., Walker, J. R., Altamentova, S., Evdokimova, E., Vedadi, M., Bochkarev, A., Savchenko, A., Glyoxylate and pyruvate are antagonistic effectors of the *Escherichia coli* IclR transcriptional regulator. J. Biol. Chem. 2007, 282, 16476-16491.

Matsuo, S., Investigation on carbon catabolite repression and co-consumption of multiple carbon sources in *Escherichia coli*. M.S. thesis, Kyushu Institute of Technology 2011.

Matsuoka, Y., Shimizu, K., Catabolite regulation analysis of *Escherichia coli* for acetate overflow mechanism and co-consumption of multiple sugars based on systems biology approach using computer simulation. J. Biotechnol. 2013, 168, 155-173.

Morita, T., El-Kazzaz, W., Tanaka, Y., Inada, T., Aiba, H., Accumulation of glucose 6-phosphate or fructose 6-phosphate is responsible for destabilization of glucose transporter mRNA in *Escherichia coli*. J. Biol. Chem. 2003, 278, 15608-15614.

Peskov, K., Mogilevskaya, E., Demin, O., Kinetic modelling of central carbon metabolism in *Escherichia coli*. FEBS J. 2012, 279, 3374-3385.

Pramanik, J., Keasling, J. D., Stoichiometric model of *Escherichia coli* metabolism: incorporation of growth-rate dependent biomass composition and mechanistic energy requirements. Biotechnol. Bioeng. 1997, 56, 398-421.

Toya, Y., Ishii, N., Nakahigashi, K., Hirasawa, T., Soga, T., Tomita, M., Shimizu, K., ^{13}C-metabolic flux analysis for batch culture of *Escherichia coli* and its *pyk* and *pgi* gene knockout mutants based on mass isotopomer distribution of intracellular metabolites. Biotechnol. Prog. 2010, 26, 975–992.

Usuda, Y., Nishio, Y., Iwatani, S., Van Dien, S. J., Imaizumi, A., Shimbo, K., Kageyama, N., Iwahata, D., Miyano, H., Matsui, K., Dynamic modeling of *Escherichia coli* metabolic and regulatory systems for amino-acid production. J. Biotechnol. 2010, 147, 17-30.

Wada, K., Toya, Y., Banno, S., Yoshikawa, K., Matsuda, F., Shimizu, H., ^{13}C-metabolic flux analysis for mevalonate-producing strain of *Escherichia coli*. J. Biosci. Bioeng. 2017, 123, 177-182.

Wolf, R. E. Jr, Prather, D. M., Shea, F. M., Growth-rate-dependent alteration of 6-phosphogluconate dehydrogenase and glucose 6-phosphate dehydrogenase levels in *Escherichia coli* K-12. J. Bacteriol. 1979, 139, 1093-1096.

Yao, R., Hirose, Y., Sarkar, D., Nakahigashi, K., Ye, Q., Shimizu, K., Catabolic regulation analysis of *Escherichia coli* and its *crp, mlc, mgsA*, *pgi* and *ptsG* mutants. Microb. Cell Fact. 2011, 10, 67.
